# Spatial transcriptome profiling by MERFISH reveals fetal liver hematopoietic stem cell niche architecture

**DOI:** 10.1038/s41421-021-00266-1

**Published:** 2021-06-29

**Authors:** Yanfang Lu, Miao Liu, Jennifer Yang, Sherman M. Weissman, Xinghua Pan, Samuel G. Katz, Siyuan Wang

**Affiliations:** 1 Department of Genetics, Yale School of Medicine, New Haven, USA; 2 Department of Biochemistry and Molecular Biology, School of Basic Medical Sciences, Southern Medical University, Guangzhou, Guangdong China; 3 Guangdong Provincial Key Laboratory of Single Cell Technology and Application, Guangzhou, Guangdong China; 4 Department of Pathology, Yale School of Medicine, New Haven, USA; 5 Department of Cell Biology, Yale School of Medicine, New Haven, USA

**Keywords:** Haematopoietic stem cells, Stem-cell niche

## Abstract

The hematopoietic stem cell (HSC) niche has been extensively studied in bone marrow, yet a more systematic investigation into the microenvironment regulation of hematopoiesis in fetal liver is necessary. Here we investigate the spatial organization and transcriptional profile of individual cells in both wild type (WT) and *Tet2*^−*/*−^ fetal livers, by multiplexed error robust fluorescence in situ hybridization. We find that specific pairs of fetal liver cell types are preferentially positioned next to each other. Ligand-receptor signaling molecule pairs such as *Kitl* and *Kit* are enriched in neighboring cell types. The majority of HSCs are in direct contact with endothelial cells (ECs) in both WT and *Tet2*^−*/*−^ fetal livers. Loss of *Tet2* increases the number of HSCs, and upregulates Wnt and Notch signaling genes in the HSC niche. Two subtypes of ECs, arterial ECs and sinusoidal ECs, and other cell types contribute distinct signaling molecules to the HSC niche. Collectively, this study provides a comprehensive picture and bioinformatic foundation for HSC spatial regulation in fetal liver.

## Introduction

Hematopoietic stem cells (HSCs) retain the potential to generate all lineages of blood cells throughout the life of the organism. Long-term HSCs arise first in the dorsal aorta of aorta–gonad–mesonephros region of mouse embryos around embryonic day 10.5 (E10.5) through an endothelial-to-hematopoietic transition^1^. On day 12 of gestation, HSCs migrate to the fetal liver through the blood circulation via the umbilical vein from the placenta. In the fetal liver, HSCs undergo a 38-fold expansion until E16^2^. Finally, HSCs migrate from the liver to the bone marrow where they maintain life-long hematopoiesis.

The microenvironment of HSCs, termed the “HSC niche”, influences fundamental properties of the HSCs, including proliferation, self-renewal, differentiation, and migration^3^. Various niches are present in different stages of development given the diversity of tissues where HSCs reside, including the yolk sac, aorta–gonad–mesonephros region, placenta, fetal liver, spleen, and bone marrow^4–6^. Distinct niches may have diverse responsibilities to meet the demand of hematopoietic cells during homeostasis and stress^7^. For example, fetal HSCs are more efficient in regenerating certain cell populations such as CD5^+^ B cells than adult HSCs^8^. Another difference is that HSCs are mostly quiescent in bone marrow^9^, whereas they actively proliferate in fetal liver^2^. However, in both settings the HSC niche tends to include endothelial cells (ECs), either as part of portal vessels in the fetal liver or arterioles in the bone marrow^10,11^. How prevalent HSCs are in direct contact with ECs is unknown. The study of HSC niches will further our understanding and treatment of various hematopoietic diseases. Because it is difficult to attain sufficient HSCs for direct clinical use, understanding a niche that supports rapid HSC expansion may offer guidance to amplify HSCs ex vivo.

Several microenvironment-dependent signaling pathways have been shown as essential in regulating the self-renewal and differentiation of HSCs in the bone marrow. These include: CXCL12/CXCR4 signaling, BMP signaling, Mpl/TPO signaling, Tie2/Ang-1 signaling, Wnt signaling, and Notch signaling^12^. Wnt and Notch are particularly complicated signaling pathways in the niche, because a large number of gene family members are involved in each pathway, and because different levels of pathway activation yield different overall effects on the HSCs and hematopoiesis^13,14^. How these signaling pathways contribute to fetal liver HSCs needs further investigation.

In addition to signaling pathways in the microenvironment, epigenetic elements also regulate HSC development, heterogeneity and proliferation. In particular, *Tet2* encodes a well-known epigenetic modifier that plays important roles in hematopoiesis. TET2 regulates DNA methylation by conversion of 5-methylcytosine (5mC) to 5-hydroxymethylcytosine (5hmC)^15,16^. Disruption of *Tet2* enhances HSC self-renewal, and leads to myeloid transformation^17,18^. In addition to cell-autonomous functions within the HSC, loss of *Tet2* increases bone marrow stromal cell (BMSC) self-renewal and proliferation capacity, and enhances their hematopoietic supportive capacity^19^. Clinically, loss-of-function mutations of *Tet2* are found in clonal hematopoiesis of indeterminate potential^20^, 26% of myelodysplasia^21^, 12% of acute myeloid leukemia, and 42% of chronic myelomonocytic leukemia^22^. Yet the effect of *Tet2* in the fetal liver niche context is only partially understood.

To better understand the function of the HSC niche, a systematic characterization of cell types and active molecular pathways within the HSC microenvironment is required. Single-cell RNA-sequencing (scRNA-seq) allows profiling the heterogeneity of gene expression in individual cells^23^. However, standard scRNA-seq requires cell dissociation, which destroys the spatial context and cell–cell interactions in the niche. Recent spatial scRNA-seq modalities detect spatial distribution of cell types, but are limited by the spatial resolution (some only partially matching the spatial scale of single cells), detection efficiency, and/or drop-out rate^24,25^. Single molecule fluorescence in situ hybridization (smFISH) is a powerful tool to map and count RNAs in situ with high spatial resolution and detection efficiency, but traditional smFISH can only measure a few RNA species at a time^26^. Multiplexed error-robust fluorescence in situ hybridization (MERFISH) is a highly multiplexed FISH method to map hundreds to thousands of RNA species at the single molecule level in single cells^27–29^. MERFISH is based on a combinatorial barcoding and error-robust encoding scheme to label numerous RNA species, and a sequential smFISH strategy to read out the barcodes, each of which is associated with different RNA species^27–29^. Here, we use MERFISH and scRNA-seq to explore hematopoiesis in mouse fetal liver and how it is altered in the absence of TET2. The experimental workflow is illustrated in Supplementary Fig. S1. Using MERFISH, we profile numerous niche factors, signaling factors, and cell type marker transcripts simultaneously at the single cell level in situ. We analyze how cells of different types are spatially organized into microenvironments, and how the cell type and the microenvironment organization jointly affect transcript profiles. Our results show that specific pairs of cell types are enriched as neighbors in the fetal liver microenvironments. Particularly, HSCs locate predominantly in endothelial niches and the majority of HSCs are in direct contact with one or several types of ECs. We further compare the HSC niches in wild type (WT) and *Tet2*^−*/*−^ fetal livers and show that loss of *Tet2* leads to an increased number of HSCs, as well as increased expression of Wnt and Notch signaling genes within the HSC niche, suggesting a causal link between the molecular and cellular changes and related pathology. In addition, we show that arterial ECs (AECs), sinusoidal ECs (SECs), and other fetal liver cell types offer distinct signaling molecules to the HSC niche.

## Results

### scRNA-seq and MERFISH identify major cell types in fetal liver

**Fig. 1:**
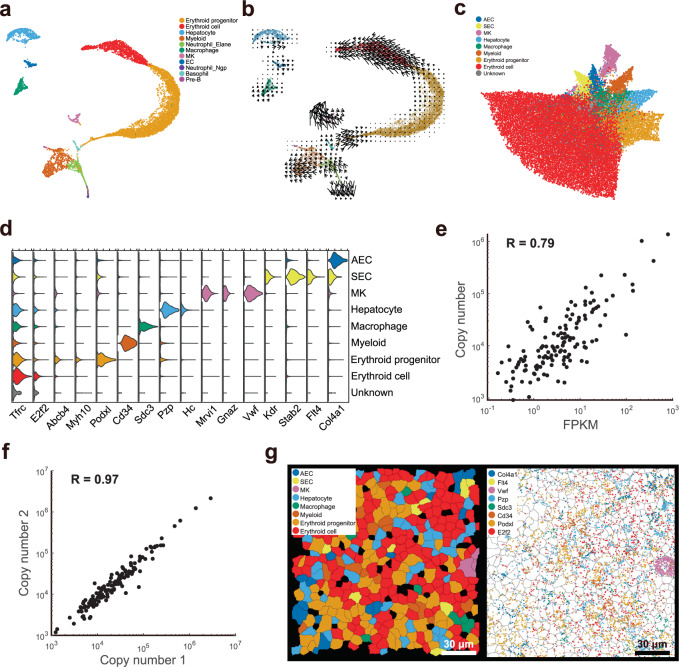
MERFISH identifies major cell types in fetal liver hematopoiesis. **a** UMAP visualization of cell clustering of 11 major cell types in the WT fetal liver by scRNA-seq (*n* = 7,635 cells). MK, megakaryocyte; EC, endothelial cell; Pre-B, Pre-B cell. **b** The observed and the extrapolated future states (arrows) are shown on the pre-defined UMAP. RNA velocity was estimated with spliced and unspliced RNA ratios in single cells. **c** UMAP plot of MERFISH data of WT fetal liver (*n* = 40,864 cells). We attained eight major cell types with MERFISH data with an extra small group of “Unknown” cells that we cannot distinguish. **d** Violin plot of example cell type markers in MERFISH data. *x* axis shows normalized abundance ranging from 0 to 0.3 for each gene. See Methods for the normalization procedure. **e** The correlation analysis of total RNA copy numbers from MERFISH imaging vs FPKM values from bulk RNA-seq for each probed RNA species. **f** The correlation analysis of total RNA copy numbers of two biological replicates of MERFISH for each probed RNA species. **g** The in situ map of identified cell types in a field of view (left panel) and the detected transcripts of example marker genes plotted in the same field of view (right panel).

To characterize the gene expression features of different cell types in E14.5 mouse fetal liver, we first identified 11 major cell types in fetal liver with scRNA-seq (Fig. 1a). Cell type assignment was based on the marker genes selected from the Mouse Cell Atlas database^30^. We performed RNA velocity analysis to derive the developmental relationship among the different cell types (Fig. 1b). RNA velocity was analyzed by calculating the ratio of spliced to unspliced mRNAs in single cells, which predicts the future state of each cell^31^. According to a two-dimensional representation of the single cell transcriptome and the direction of the arrows in our RNA velocity map, erythroid cells, erythroid progenitors, myeloids, basophils, and neutrophils were connected in a branched development trajectory, while megakaryocytes (MKs) and macrophages were separated from the above mentioned cells; erythroid progenitors differentiated into erythroid cells; and neutrophils were separated into two main subtypes (Neutrophil_Elane and Neutrophil_Ngp) that appear to be differentiated from a common progenitor (Fig. 1b).

We further analyzed the gene ontology (GO) (biological processes) of differentially expressed genes (DEGs) in myeloid cells, MKs, erythroid progenitors, erythroid cells, HSCs, and ECs, in which each cell type was compared to the other cell types (Supplementary Fig. S2). GO analysis of DEGs showed that myeloid cells, as expected, mainly committed themselves to defense response and immune response (Supplementary Fig. S2a); DEGs of MKs showed wound healing, hemostasis, and platelet activation functions (Supplementary Fig. S2b); DEGs of erythroid progenitors were enriched for heme metabolic and biosynthetic processes (Supplementary Fig. S2c); erythroid cells expressed genes related to erythrocyte development and homeostasis (Supplementary Fig. S2d); HSCs expressed genes that regulate the immune system, hematopoietic or lymphoid organ development and hemopoiesis (Supplementary Fig. S2e); and DEGs of ECs were enriched with circulatory system development and vasculature/blood vessel development functions (Supplementary Fig. S2f).

To analyze the distinct fetal liver cell types and their gene expression profiles in real space, we designed a MERFISH probe library that targets 45 cell-type marker genes selected from the scRNA-seq data and 95 niche-associated factors or signaling/marker genes from literature (Supplementary Table S1). We segmented cells with oligo-conjugated wheat germ agglutinin labeling of cell membrane components (Supplementary Fig. S3). The raw images of MERFISH showed distinct RNA foci (Supplementary Fig. S4). The single-cell RNA copy numbers of 132 selected genes (after excluding eight low quality RNA species; see Materials and Methods) allowed us to identify eight major cell types using the fetal liver MERFISH data (Fig. 1c and Supplementary Fig. S5). Note that MERFISH captures a more accurate representation of the proportion of erythroid cells compared to scRNA-seq (Supplementary Fig. S6), which commonly uses red blood cell lysis buffer. The cell types were identified based on the marker gene expression levels (Fig. 1d). Population-level RNA copy numbers measured by MERFISH were highly correlated with FPKM values from bulk RNA sequencing, with a correlation coefficient of 0.79 (Fig. 1e). Biological replicates of MERFISH measurements showed a correlation coefficient of 0.97 (Fig. 1f). These high correlations validated the quality of the MERFISH data. The identified eight major cell types were mapped in situ back onto the tissue sections, revealing their spatial organization (Fig. 1g).

### Spatial proximity of pairs of cell types in WT and *Tet2*^*−*^^*/*−^ fetal liver

**Fig. 2:**
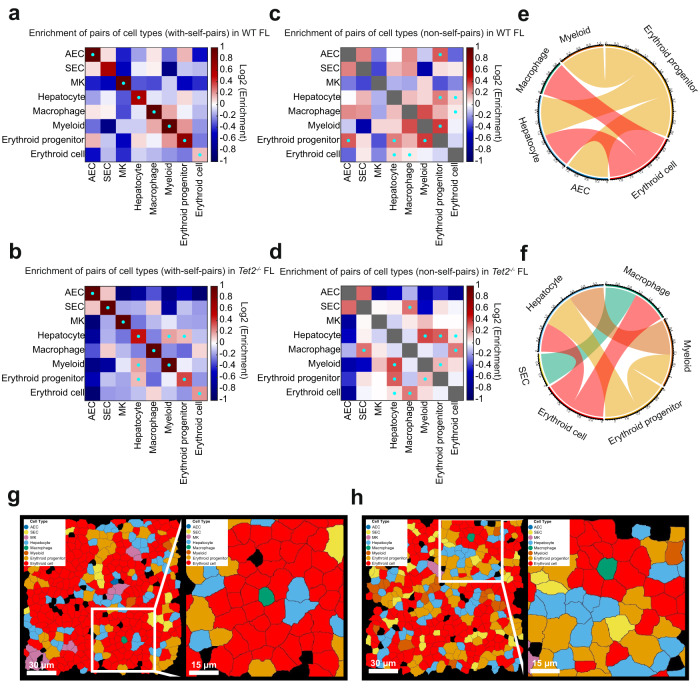
Enrichment of pairs of cell types in spatial proximity in WT and *Tet2*^−*/*−^ fetal livers. **a**, **b** The enrichment folds of pairs of cell types in spatial proximity with self-pairs in WT (**a**) and *Tet2*^−*/*−^ (**b**) fetal livers. **c**, **d** The enrichment folds of pairs of cell types in spatial proximity without self-pairs in WT (**c**) and *Tet2*^−*/*−^ (**d**) fetal livers. The color bars are scaled to ±1 (**a**–**d**) with red indicating enrichment and blue indicating depletion. Cyan dots represent *P* values < 0.01 for positive enrichment with false discovery rate correction. **e**, **f** The chord diagram plots of significantly enriched pairs in WT (**e**) and *Tet2*^−*/*−^ (**f**) fetal livers. **g**, **h** MERFISH cell type plots of a central macrophage (green) in an erythroid island (red). Cell types were determined using MERFISH data and mapped in situ (left panels). A fourfold magnified image of the region in the white box is shown on the right. AEC, arterial endothelial cell; SEC, sinusoidal endothelial cell; MK, megakaryocyte.

To better understand the spatial organization of cells in fetal liver, we analyzed the enrichment of pairs of cell types in spatial proximity in WT and *Tet2*^−*/*−^ fetal livers (Fig. 2). Here, we defined two cells that are less than 20-μm apart as in a neighborhood, and calculated the probability of cell type pairs in neighborhoods. We then calculated the enrichment of cell type pairs in spatial proximity by normalizing this probability to the control probability based on random pairing (see Materials and Methods). The most prominent feature from this spatial proximity analysis was that cells tended to aggregate with cells of the same type in situ (Fig. 2a, b). Due to the highly enriched spatial self-clustering, neighboring pairs of different cell types were mostly depleted when self pairs are calculated together with non-self pairs. To further capture enrichment just between non-self pairs, we removed the self pairs from the analysis (Fig. 2c–f). This allowed us to capture several enriched pairs of different cell types in spatial proximity. In both WT (Fig. 2c, e) and *Tet2*^−*/*−^ (Fig. 2d, f) fetal livers, macrophages were paired with erythroid cells; hepatocytes and myeloid cells were paired with erythroid progenitors; hepatocytes were also paired with erythroid cells. In addition, erythroid progenitors were paired with AECs in WT fetal liver (Fig. 2c, e and Supplementary Fig. S7a); macrophages were paired with SECs, and hepatocytes with myeloid cells in *Tet2*^−*/*−^ fetal liver (Fig. 2d, f and Supplementary Fig. S7b). In summary, erythroid progenitors lost a neighboring relationship with AECs, myeloids gained a neighboring relationship with hepatocytes, and macrophages gained a neighboring relationship with SECs in *Tet2*^−*/*−^ fetal liver (Supplementary Fig. S8). Previous studies have shown that macrophages tended to be surrounded by erythroid cells forming an erythroblastic island and to regulate the differentiation, proliferation, and clearance of erythroid cells^32^, which is consistent with our observation (Fig. 2g, h and Supplementary Fig. S7c). These results indicate that fetal liver cell types are spatially paired in a non-random fashion, suggesting cell-cell communications.

### Expression of known niche factors in fetal liver cell types

**Fig. 3:**
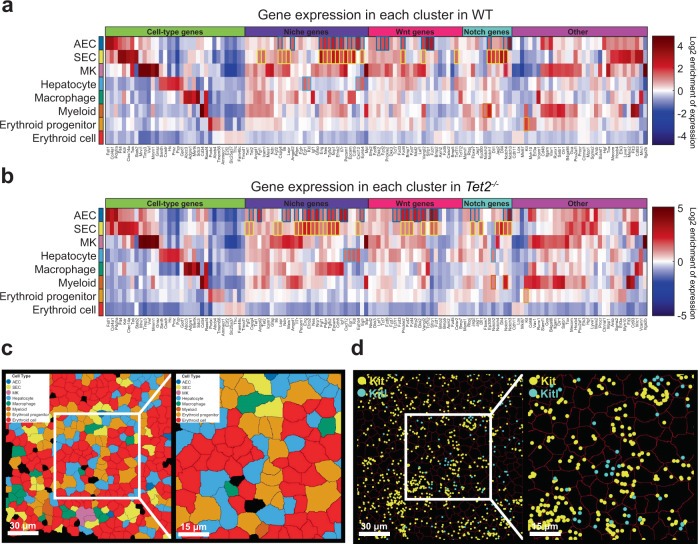
Gene expression enrichment in different cell types in WT and *Tet2*^−*/*−^ fetal livers. **a**, **b** Gene expression enrichment profiles of probed genes in different cell types in WT (**a**) and *Tet2*^*−*^^*/*−^ (**b**) fetal livers. All genes used in the MERFISH analysis are shown along the x-axes. Log_2_ enrichment of expression for each gene is shown by a red/blue color scale (on the right) with darker red indicating a stronger enrichment and darker blue a stronger depletion. The niche genes (dark violet), Wnt genes (deep pink), Notch genes (cyan), and other genes (magenta) were ordered based on hierarchical clustering in each category. All the log_2_ enrichment values of the highlighted niche/Wnt/Notch genes are larger than 1. AEC, arterial endothelial cell; SEC, sinusoidal endothelial cell; MK, megakaryocyte. **c**, **d** An example of *Kitl* positive hepatocytes with nearby *Kit* positive erythroid progenitors. Cell types were determined using MERFISH data and mapped in situ (**c**). Individual mRNA molecules from MERFISH were plotted on top of the segmented cell image for *Kitl* (cyan dots) and *Kit* (yellow dots) (**d**). The right panels in **c** and **d** are fourfold magnified views of the white boxed regions in the left panels.

To explore the expression of various HSC niche factors in different cell types, we calculated the enrichment of gene expression in different cell types over the population-averaged expression profile in both WT (Fig. 3a) and *Tet2*^−*/*−^ (Fig. 3b) populations. As expected, cell type markers were enriched in the corresponding cell types. For example, megakaryocyte markers *Gnaz*, *Mrvi1*, *Vwf*, *Timp3*, and *Mmrn1* were enriched in MKs. We found many known HSC niche genes such as *Fgf1*, *Icam1*, *Cspg4*, *Il6*, *Lepr*, *Angptl2*, *Eng*, *Nes*, *Tgfb2*, *Nrp1*, *Efnb2*, *Il7r*, *Pecam1*, *Epcam*, *Cdh5*, *Cxcl12*, and *Ephb4*, Wnt genes such as *Dkk2*, *Fzd2*, *Prickle2*, *Fzd4*, *Vangl2*, *Sfrp1*, *Fzd1*, and *Tcf7l1*, and Notch genes such as *Notch1*, *Dll1*, *Jag2*, *Dll4*, *Notch4*, and *Notch3* were enriched in WT AECs or SECs (Fig. 3a); and niche genes such as *Fgf2*, *Cspg4*, *Angptl2*, *Il6*, *Lepr*, *Ndn*, *Meis1*, *Il7r*, *Pecam1*, *Eng*, *Efnb2*, *Nes*, *Nrp1*, *Fgf1*, *Pdpn*, *Tgfb2*, *Epcam*, *Cdh5*, *Igf1*, *Cxcl12*, *Ephb4*, and *Egfr*, Wnt genes such as *Tcf7l1*, *Fzd3*, *Prickle2*, *Fzd2*, *Fzd4*, *Sfrp2*, *Dkk2*, *Vangl2*, *Sfrp1*, and *Fzd1*, Notch genes such as *Jag2*, *Jag1*, *Dll1*, *Notch4*, *Dll4*, *Notch1*, and *Notch3* were enriched in *Tet2*^−*/*−^ AECs or SECs (Fig. 3b). This suggests that ECs are particularly important for supporting the HSC niche. To further analyze the cell–cell communications in the HSC niche, we performed fluorescence-activated cell sorting (FACS) to enrich the HSCs and integrated scRNA-seq datasets of sorted HSCs and of whole fetal liver (Supplementary Fig. S9a). A cellphone analysis^33^ of ligand/receptor expression using our scRNA-seq data showed that HSCs were potentially closely communicating with ECs (Supplementary Fig. S10a). Several well characterized Wnt and Notch ligand/receptor pairs underlie this potential interaction (Supplementary Fig. S10b).

Based on the gene expression enrichment analyses, some niche genes were also enriched in non-EC cell types. For instance, *Cxcl12*, previously reported as crucial for HSC maintenance in bone marrow^34–36^, was enriched in both AECs and hepatocytes. *Kitl* (*Kit* ligand), which supports HSC expansion in fetal liver^37^, was enriched in hepatocytes. *Egr1*, known to be expressed in HSCs and to coordinate HSC division and migration^38^, was enriched in hepatocytes (Fig. 3a, b). A full list of gene expression enrichments in different cell types is provided in Supplementary Table S2. One explanation to these observations is that the different cell types offer the corresponding supportive molecules for HSC regulation. However, these findings also raise the possibility that these niche genes may have additional non-HSC functions within the corresponding cell types. Indeed, as we found that hepatocytes were paired with erythroid progenitors in space (Fig. 2c–f), *Kitl* and *Kit* were enriched in hepatocytes and erythroid progenitors, respectively (Fig. 3a, b). In situ maps further confirmed that *Kitl-*positive hepatocytes were usually near *kit*-positive erythroid progenitor cells (Fig. 3c, d), suggesting ligand–receptor communication between the two cell types.

We also observed that in WT fetal liver *Notch1* was enriched in AECs, SECs, and myeloid cells; *Notch2* was enriched in myeloid cells; *Notch3* was enriched in AECs; *Notch4* was enriched in AECs, SECs, and MKs (Fig. 3a). In *Tet2*^−*/*−^ fetal liver, *Notch1* was enriched in AECs, SECs, and myeloid cells; *Notch2* was enriched in hepatocytes and myeloid cells; *Notch3* was enriched in AECs and SECs; *Notch4* was enriched in SECs and MKs (Fig. 3b). These suggest that different Notch genes may have cell type-specific functions or cell type biases.

### Identification of HSCs and characterization of the HSC niches in WT and *Tet2*^*−*^^*/*−^ fetal livers by MERFISH

Although the cellphone analysis of scRNA-seq data suggested that ECs communicate with HSCs (Supplementary Fig. S10a), this sequencing-based analysis only provides the potential molecular interactions, but not the direct spatial information. Thus, it cannot directly measure the frequency at which ECs are physically associated with HSCs in space and the molecular and cellular compositions of the HSC niche. To tackle these questions, we set out to identify HSCs in the MERFISH data. To select the specific markers of HSCs, we identified cell types based on known marker genes from our integrated HSC and whole fetal liver scRNA-seq datasets (Supplementary Fig. S9b). We found that *Mecom* was specifically and highly expressed in the HSC groups (Supplementary Fig. S9c). In addition, *Kit* was expressed in HSCs (Supplementary Fig. S9c). This agrees with previous studies showing that *Mecom* was expressed in 60% of mouse long-term HSCs and at high levels in human hematopoietic stem and progenitor cells^39,40^, and was regarded as critical for long-term HSC function^41^. *Kit* was found essential for quiescent HSC maintenance^42^, and mice lacking *Kit* showed hematopoietic defects and perinatal death^43^. Thus we defined HSCs as positive for *Kit* and *Mecom* expression, and used this criteria to identify HSCs in MERFISH data (Supplementary Fig. S5).

**Fig. 4:**
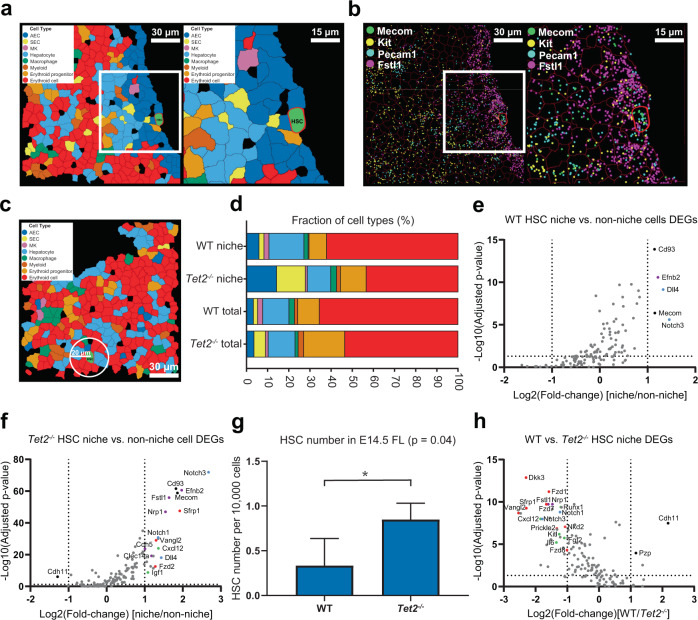
Features of the HSC niche by MERFISH analysis. **a** An example of an HSC embedded in a vascular wall seen by MERFISH imaging, cell types were determined and mapped in situ (left panel). **b** Individual mRNA molecule FISH signals were visualized for *Mecom* (green dots), *Kit* (yellow dots), *Fstl1* (magenta dots), and *Pecam1* (cyan dots) in situ. **c** An example of an HSC niche identified by MERFISH imaging. The niche area is marked by a white circle with a 20-μm radius. The cell boundaries were segmented based on the membrane labeling with wheat germ agglutinin (WGA). The right panels in **a** and **b** are fourfold magnified views of the white boxed regions in the left panels. The HSC boundary is shown in red. **d** The cell type percentages of the HSC niche and of the total cell population were calculated for WT and *Tet2*^−*/*−^ fetal livers (WT niche: *n* = 254 cells; *Tet2*^−*/*−^ niche: *n* = 550 cells; WT overall: *n* = 112,392 cells; *Tet2*^−*/*−^ overall: *n* = 137,628 cells). The cell type color scheme of **d** is the same as **a** and **c**. AEC, arterial endothelial cell; SEC, sinusoidal endothelial cell; MK, megakaryocyte. **e**, **f** The DEGs between the HSC niche and non-niche in WT (**e**) and *Tet2*^−*/*−^ (**f**) fetal livers. **g** HSCs were significantly increased in *Tet2*^*−*^^*/*−^ (*n* = 3 biological replicates, 0.848 ± 0.183) compared with WT (*n* = 4 biological replicates, 0.334 ± 0.301) in E14.5 fetal livers (unpaired *t*-test with Welch’s correction, *P* = 0.04). **h** The DEGs between the WT and *Tet2*^−*/*−^ HSC niches. In **e**, **f**, and **h**, the horizontal dotted line represents an adjusted *P* value = 0.05 using a Wilcoxon rank-sum test with false discovery rate correction. The vertical lines represent a fold change = 2. Blue dots are Notch pathway genes, red dots are Wnt pathway genes, purple dots are vasculature genes, and black dots are other genes. All the labeled dots are reproducible significant genes.

To characterize the HSC niche in situ, we measured the cell type compositions and gene expression features in HSC niches in their native spatial context by MERFISH. Approximately 53% and 75% of HSCs in WT and *Tet2*^−*/*−^ fetal livers, respectively, were in direct contact with ECs, based on MERFISH imaging. However, this may be an underestimate given that our tissue section largely contained a single layer of cells, and an EC might be in contact with an observed HSC but be excluded from the section. In some instances, HSCs were identified in large regions consisting of many AECs, likely forming a central vein (Fig. 4a, b). We defined an HSC niche as a 20-μm-radius area centered at an HSC (Fig. 4c). A comparison between the proportion of ECs within HSC niches vs the proportion of ECs in the entire cell population showed an enrichment of ECs in the HSC niche (Fig. 4d). Specifically, 8% of the cells in the WT HSC niche were ECs compared to 5% overall (21/254 vs 5,876/112,392, *P* = 0.0422). This enrichment of ECs was even more dramatic for the *Tet2*^−*/*−^ HSC niches, where 28% of the cells were ECs compared to 9% of cells being ECs in the entire population (153/550 vs 12,309/137,628, *P* < 0.001). In addition, erythroid progenitors were increased in the overall population in the *Tet2*^−*/*−^ fetal liver in comparison to WT (19% vs 10%, 26,627/137,628 vs 11,460/112,392, *P* < 0.001).

We further investigated the DEGs in HSC niches vs outside of the niches. The endothelial arterial marker, Ephrin-B2 (*Efnb2*) showed increased expression in both WT and *Tet2*^−*/*−^ HSC niches in comparison to cells outside of the niches (Fig. 4e, f). Other endothelial markers highly expressed in the *Tet2*^−*/*−^ HSC niche included *Fstl1*, *Nrp1*, *Cdh5*, and *Clec14a* (Fig. 4f). The Notch signal pathway genes *Notch3* and *Dll4* were significantly upregulated in both WT and *Tet2*^*−*^^*/*−^ HSC niches (Fig. 4e, f).

Based on the MERFISH results, *Tet2*^*−*^^*/*−^ mice exhibited an increased number of HSCs when compared with WT (Fig. 4g). This is in agreement with some prior studies showing that LSK and CD150^+^ HSCs were increased in extramedullary hematopoiesis of adult *Tet2*^−*/*−^ mice^18^, but does not support some other studies reporting that HSC abundance in *Tet2*^−*/*−^ fetal livers did not change^17^. The increased HSC number we observed in *Tet2*^−*/*−^ fetal livers may be caused by changes in the HSC niche.

We next compared the DEGs in the HSC niches in *Tet2*^−*/*−^ vs WT fetal livers (Fig. 4h). Most notably, several Wnt signaling pathway related genes were significantly upregulated in the *Tet2*^−*/*−^ HSC niche. This included canonical Wnt Frizzled (Fzd) receptors *Fzd1*, *Fzd7*, and *Fzd8*. In contrast, *Fzd2*, *Fzd3*, *Fzd4*, and *Fzd5*, the downstream signaling gene *Axin2*, and the responsive transcription factors *Lef1*, *Tcf7*, *Tcf7l1*, and *Tcf7l2* were not increased. The non-canonical Wnt signaling genes *Vangl2* and *Prickle2* also showed increases in expression, but not *Celsr2*. In addition to Wnt signaling agonists, other upregulated genes include Wnt antagonists, such as *Nkd2* and *Sfrp1*, but not *Sfrp2*. Interestingly, the most significantly upregulated gene in the *Tet2*^−*/*−^ HSC niche was a member of the dickkopf family, *Dkk3*, while there was no enrichment of *Dkk2* expression. Whereas DKK2 binds to LRP5/6 and inhibits the interaction of Wnt to the Frizzled receptors^44^, DKK3 is the most divergent member of the DKK family and does not bind to LRP5/6^45^. Rather, both positive and negative roles have been shown for DKK3 in Wnt signaling^45,46^.

In addition to Wnt signaling, several other genes important for cell proliferation were also upregulated in the *Tet2*^−*/*−^ HSC niche in comparison to the WT HSC niche, including *Notch1*, *Notch3*, *Cxcl12*, *Kitl*, *Fgf2*, and *Il6* (Fig. 4h). Notch signaling has been shown to increase stem cell numbers in mouse bone marrow^47,48^. *Cxcl12* is critical for HSC maintenance: HSCs were reduced in number and became more quiescent in the absence of CXCL12-abundant reticular cells^34–36^. KIT-ligand (*Kitl*) induces HSC maintenance in the bone marrow^49^ and contributes to HSC expansion in the mouse fetal liver^37^. FGF-2 stimulates HSC proliferation in vivo through *Kitl* signaling and stromal cell expansion^50^. *Il6* signaling promotes HSPC proliferation in mice^51^, and has a combinatorial effect with Notch activation on hematopoietic cells^52^. In summary, our results suggest that loss of *Tet2* leads to an increase of HSCs in fetal liver due to the upregulation of Wnt, Notch, and other niche factors that support HSC proliferation.

### Distinct signaling compositions of AECs and SECs in HSC niches

**Fig. 5:**
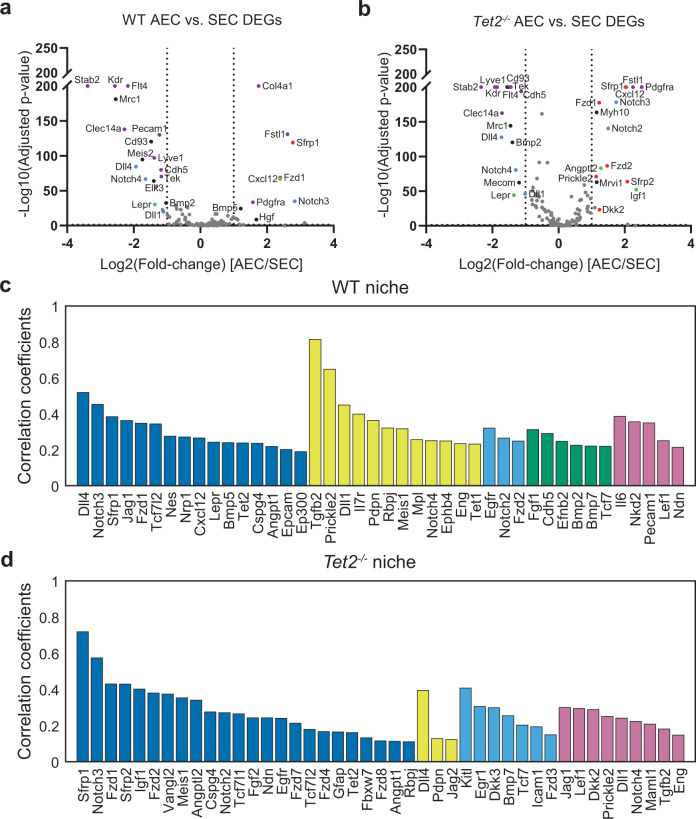
AEC and SEC contribute different molecules to WT and *Tet2*^*−*^^*/*−^ HSC niche. **a**, **b** The DEGs of AECs vs SECs in WT (**a**) and *Tet2*^−*/*−^ (**b**) fetal livers by MERFISH. The horizontal dotted line represents an adjusted *P* value = 0.05 using a Wilcoxon rank-sum test with false discovery rate correction. The vertical lines represent a fold change = 2. Blue dots are Notch pathway genes, red dots are Wnt pathway genes, green dots are niche factors, purple dots are vasculature genes, and black dots are other genes. All the labeled dots are reproducible significant genes. **c**, **d** Correlation coefficients of niche genes with cell type markers in WT (**c**) and *Tet2*^−*/*−^ (**d**) HSC niches. The genes listed in **c** and **d** all showed significant correlations (*R* > 0.1, *P* < 0.05) with the corresponding cell type markers (dark blue = AEC, yellow = SEC, light blue = hepatocyte, green = macrophage, pink = megakaryocyte). The *P* values were corrected using false discovery rate.

Previous studies suggested that both AECs and SECs were essential for HSC regeneration and maintenance^5,53^. To further identify the specific roles of AECs and SECs, we first analyzed their DEGs (Fig. 5a, b). In both WT and *Tet2*^−*/*−^ fetal livers, SECs expressed specific endothelial markers such as *Flt4*, *Stab2*, *Kdr*, *Cdh5*, *Clec14a*, *Tek*, and *Lyve1*, whereas AECs specifically expressed other endothelial markers such as *Fstl1* and *Pdgfra*. In addition, SECs from both WT and *Tet2*^−*/*−^ fetal livers expressed the Notch pathway genes *Notch4*, *Dll1* and *Dll4*, and the niche factor *Lepr*, whereas AECs expressed *Notch3* and *Cxcl12*.

The analysis above included AECs and SECs inside and outside of HSC niches. To explore the co-expression of various niche factors among cells within HSC niches, we calculated the correlation coefficients of pairs of genes co-expressed in the same HSC niche cells in the WT (Fig. 5c) and *Tet2*^−*/*−^ (Fig. 5d) fetal livers using the MERFISH data. Note the scRNA-seq procedure removes a large population of ECs in fetal liver (Supplementary Fig. S6), and has lower cell number throughput than MERFISH in general (Fig. 1a with 136 ECs out of 7,635 cells in total and Fig. 1c with 2,546 ECs out of 40,864 cells in total). Besides, to analyze the gene co-expression in niche requires the cellular spatial distribution information, which is not preserved by scRNA-seq, but by MERFISH. We found that in WT HSC niches, niche genes *Nes*, *Nrp1*, *Cxcl12*, *Lepr*, *Bmp5*, *Tet2*, *Cspg4*, *Angpt1*, and *Epcam*, Wnt and Notch signaling genes *Dll4*, *Notch3*, *Sfrp1*, *Jag1*, *Fzd1*, *Tcf7l2*, and *Ep300* were correlated with AEC, but not SEC, marker genes. In contrast, niche genes *Tgfb2*, *Il7r*, *Pdpn*, *Meis1*, *Mpl*, *Ephb4*, *Eng*, and *Tet1*, and Wnt and Notch signaling genes *Prickle2*, *Dll1*, *Rbpj*, and *Notch4* were correlated with SEC, but not AEC, marker genes (Fig. 5c). In *Tet2*^*−*^^*/*−^ HSC niches, niche genes *Igf1*, *Meis1*, *Angptl2*, *Cspg4*, *Fgf2*, *Ndn*, *Egfr*, *Gfap*, *Tet2*, and *Angpt1*, and Wnt and Notch signaling genes *Sfrp1*, *Notch3*, *Fzd1*, *Sfrp2*, *Fzd2*, *Vangl2*, *Notch2*, *Tcf7l1*, *Fzd7*, *Tcf7l2*, *Fzd4*, *Fbxw7*, *Fzd8*, and *Rbpj* were correlated with AEC marker genes; niche gene *Pdpn*, and Notch signaling genes *Dll4* and *Jag2* were correlated with SEC marker genes (Fig. 5d). Note that the expression of *Tet2* mRNA in the *Tet2*^−*/*−^ mice is expected based on the knockout design that removes only exons 8 through 11^54^. These results indicate that AEC and SEC may contribute different niche molecules to HSC niche in fetal liver.

We also observed several niche or signaling genes correlated with markers of other cell types in the HSC niche. For example, in WT HSC niches, *Egfr*, *Notch2*, and *Fzd2* were co-expressed with hepatocyte marker genes; *Fgf1*, *Cdh5*, *Efnb2*, *Bmp2*, *Bmp7*, and *Tcf7* were co-expressed with macrophage marker genes; and *Il6*, *Nkd2*, *Pecam1*, *Lef1*, and *Ndn* were co-expressed with megakaryocyte marker genes (Fig. 5c). In *Tet2*^−*/*−^ HSC niches, *Kitl*, *Egr1*, *Dkk3*, *Bmp7*, *Tcf7*, *Icam1*, and *Fzd3* were co-expressed with hepatocyte marker genes; *Jag1*, *Lef1*, *Dkk2*, *Prickle2*, *Dll1*, *Notch4*, *Maml1*, *Tgfb2*, and *Eng* were co-expressed with megakaryocyte marker genes (Fig. 5d). A full list of correlations between niche/signaling genes and cell type marker genes is provided in Supplementary Table S3. These results suggest that cell types other than ECs may also provide additional supporting molecules to the HSC niche.

## Discussion

In summary, using MERFISH and scRNA-seq we showed that fetal livers display intricate cellular and molecular organizations, indicating potential cell–cell communications and collaborations. HSCs locate predominantly in endothelial niches and the majority of HSCs directly contact one or more ECs (AECs and/or SECs). AECs and SECs possess different molecules and may establish distinct functions for HSCs. Other cell types may also contribute to HSC regulation, as well as regulating each other (e.g., hepatocytes may regulate erythroid progenitor development). Loss of *Tet2* led to an increased number of HSCs and an increased expression of Wnt and Notch signaling genes as well as other niche factors within the HSC niches, which potentially drives the HSC expansion.

Single cell in situ transcriptomics provides a valuable tool to answer many long-standing questions in biology that require an appreciation of tissue architecture. Previous studies using high throughput RNA smFISH-like techniques, including STARmap^55^, seqFISH+^56^, or MERFISH^27–29^, often dealt with tissues with well-separated cell bodies (e.g., brain tissue) or cell lines grown in vitro at densities that minimized cell–cell contact. Under these conditions, it is possible to perform cell segmentation by Nissl staining or RNA density. In contrast, the fetal liver contains many closely packed cells. Thus, to effectively detect cell boundary and segment single cells in fetal liver tissue sections is challenging. In our study, we used WGA, which marks the plasma membrane of eukaryotic cells by binding to the *N*-acetylglucosamine of glycoproteins and glycolipids^57^. Moreover, we attached WGA to an oligonucleotide to facilitate the visualization of WGA binding pattern in a manner compatible with MERFISH tissue clearing, and successfully segmented single cells in fetal liver tissue sections (Supplementary Fig. S3). We expect this procedure to be widely applicable to many tissue types, and to enable MERFISH single cell analyses of tissues with tightly packed cells.

Consistent with prior work^10^, we found that fetal liver HSCs highly colocalize with ECs. Extending Khan et al.’s observation that *Efnb2*-positive portal vessels were abutting Nestin^+^ cells, which were enriched for fetal liver HSC niche and expansion factors^10^, we discovered that *Efnb2* is more highly expressed in the fetal liver HSC niche than outside of the niche. Not elucidated in previous studies, we revealed that the majority of HSCs are directly touching at least one EC. Furthermore, we demonstrated that both AECs and SECs can be present in the HSC niches, with differences in transcripts enriched in these two cell types. *Dll4*, in particular, is enriched in the fetal liver HSC niche compared to non-niche cells (Fig. 4e, f). This is consistent with a prior report that vascular DLL4 acted as a key Notch ligand that controlled early hematopoietic differentiation^58^ and that Notch signaling promoted stem cell self-renewal and inhibited differentiation^47^. Interestingly *Dll4* is associated with AECs in WT niche (Fig. 5c), but with SECs in *Tet2*^−*/*−^ niche (Fig. 5d). This switch may underlie the changed HSC behavior upon *Tet2* knockout. *Fzd1*, which is associated with AECs but not with SECs (Fig. 5c, d), is a specific receptor of *Wnt3a* which is known to be particularly important for regulating HSC fate^59,60^. This suggests a specific importance of AECs to provide *Fzd1* in the niche. Experimental validation of our findings will require the development of AEC- and SEC-specific transgenic Cre lines to specifically knock out the niche factor genes from the appropriate cell population to observe their influence on HSC functions. We also illustrated the expression of other genes known to influence HSCs as being correlated with specific cell types. Taken together, our MERFISH findings suggest that many different types of cells contribute various signaling components to the HSC niche.

Given the importance of the epigenetic modifier *Tet2* to HSC development, we evaluated the HSC niche of *Tet2*^−*/*−^ fetal liver. Kunimoto et al. showed that disruption of *Tet2* enhanced the self-renewal and long-term repopulating capacity of fetal liver HSCs in vivo^17^. We found increased numbers of HSCs by MERFISH in the *Tet2*^*−*^^*/*−^ fetal liver, which can be explained by both HSC cell-autonomous and non-cell-autonomous mechanisms. TET2 is an epigenetic regulator responsible for the hydroxylation of 5mCs to 5-hmCs, leading to DNA demethylation^15,61,62^. Loss of TET2 leads to both direct and indirect changes in gene expression^63,64^. Use of conditional knockouts and competitive bone marrow transplantation assays has shown that *Tet2* deletion augments the hematopoietic stem/progenitor cell population in a cell-autonomous manner^18,54,65,66^. In addition, loss of *Tet2* from bone marrow mesenchymal stromal cells also augments BMSC self-renewal and proliferation, which enhances their hematopoietic supportive capacity^19^. Similarly, loss of *Tet2* leads to an increase of the EC population in fetal liver (Fig. 4d), which may promote HSC expansion in a non-cell-autonomous manner. Therefore, *Tet2* deficiency may affect HSC functions in cell-autonomous and non-cell-autonomous manners.

Khan et al. suggested that WT HSCs did not expand by increasing *Kitl*, *Angptl2*, or *Igf2* in Nestin^+^ HSC-niche cells, because these factors were expressed at a similar level at E12, E13, and E14.5 in fetal liver^10^. However, in the *Tet2*^−*/*−^ setting, we found that HSC expansion factors such as *Cxcl12*, *Kitl*, *Fgf2*, and *Il6* are increased when compared with WT fetal liver. In addition, we observed several Wnt and Notch signaling genes were strongly enriched in the *Tet2*^−*/*−^ HSC niche compared to non-niche cells and compared to the WT HSC niche. The complex and nuanced native microenvironment that we elucidated with MERFISH is difficult to recapitulate by in vitro coculture systems. We look forward to the generation of advanced in vitro systems where pure HSC and niche cells can be precisely isolated and cultured in order to help functionally test some of the niche interactions learned by MERFISH. Deletion of each of the identified niche factor genes in the *Tet2* null setting would be necessary to validate their influence on fetal liver HSC functions. In addition, we report that *Kitl*-positive hepatocytes are usually adjacent to *Kit*-positive erythroid progenitor cells, which suggests ligand-receptor communications between the two cell types. This observation of a potential hematopoietic signaling function of hepatocytes is consistent with previous studies that *Kitl*-expressing fetal liver hepatoblasts support the expansion of *Kit*-expressing HSCs and potential survival or expansion of hematopoietic progenitors^37^. Functional data also show that hepatoblast-depleted mouse embryos display a decrease in *Kitl* gene expression and a reduction of the *Kit*^*+*^ HSCs and hematopoietic progenitors^67,68^. Previous reports also show that hepatocyte growth factors can directly control erythroid progenitor proliferation and differentiation^69^. Using MERFISH to further refine the gene expression map of the HSC microenvironment under different physiological or pathological conditions will improve our understanding of HSC expansion and renewal and of ways to manipulate them.

## Materials and methods

### Animal preparation and tissue sectioning

E14.5 pregnant female C57BL/6 WT and *Tet2*^−*/*−^ mice were used for fetal liver preparation. All mice were maintained under 12 h light/12 h darkness cycles at 22 °C. All animal experiments were approved by the Institutional Animal Care and Use Committee of Yale University. Pregnant females were euthanized by isoflurane inhalation and cervical dislocation. E14.5 fetal livers were dissected from embryos in cold dulbecco’s phosphate-buffered saline (DPBS) and embedded in Tissue-Tek Cryomold (VWR, 25608-916) with optimal cutting temperature compound (Tissue-Tek O.C.T.; VWR, 25608-930). Frozen tissue blocks were stored at −80 °C before cryosection. E14.5 fetal liver frozen blocks were equilibrated to −15 °C for 1 h and then cryosectioned into 10-μm-thick slices using a Leica CM1950 machine at −15 °C. Tissue sections were post-fixed immediately with 4% PFA (Electron Microscopy Sciences, 15700) in DPBS while on salinized and poly-L-lysine (Millipore, 2913997) treated coverslips (Bioptechs, 40 mm Coverslip #1.5Thick) for 20 min at room temperature. After washing out the PFA with DPBS, tissue sections were stored in 100% ethanol at −20 °C. Data were collected from four WT fetal livers at E14.5 and two *Tet2* knockout fetal livers at E14.5.

### MERFISH probe design and construction

Primary probes of MERFISH were designed using the 16-bit Hamming-distance-4 code with 140 barcodes as previously described^27–29^. The 140-gene library contained 45 cell type marker genes from our 10× Genomics single cell sequencing data of E14.5 fetal liver and 95 HSC niche-associated genes from the literature. We chose 98 of the library gene isoforms that were long enough to construct 48 target regions using a non-overlap design and 42 of the library gene isoforms with an overlap design as previously published^70^. Each MERFISH oligo had a 30-nt targeting region, three 20-nt readout regions, and two 20-nt priming regions. The template oligonucleotide sequences for MERFISH were listed in Supplementary Table S4. The gene FPKM values of MERFISH library genes ranged from 0.16 to 794 based on matched bulk RNA-sequencing.

### Primary probe synthesis

The primary probes were constructed from a complex oligonucleotide pool that was ordered from CustomArray, Genscript^27–29^. Primary probes were synthesized via limited cycle PCR to create the templates for in vitro transcription (The forward PCR primer (with T7 promoter) sequence is GCCGTACGGATAATACGACTCACTATAGGG GCGTCGTTATGGTGCAACGT, and the reverse PCR primer sequence is TTGTCGCACGTTCGGTGTCG). These templates were converted into RNA through in vitro transcription, then converted back to single stranded DNA using reverse transcription (The reverse transcription primer sequence is TTGTCGCACGTTCGGTGTCG). RNA templates were removed from the DNA oligos via alkaline hydrolysis. Finally, the DNA oligos were purified through a Zymo column and concentrated via vacuum drying.

### Silanization of coverslips

We coated the coverslips with a silane layer containing an allyl moiety to stabilize a polyacrylamide (PA) film introduced in the later “Tissue embedding and clearing” step^29^. Briefly, the coverslips were cleaned in a 1:1 mixture of 37% (vol/vol) HCl and methanol for 30 min at room temperature. Then, coverslips were rinsed in deionized water three times and in 70% ethanol once. Coverslips were dried in a 70 °C oven before being immersed in 0.1% (vol/vol) triethylamine (Millipore, TX1200-11) and 0.2% (vol/vol) allytrichlorosilane (Sigma, 107778-5 G) in chloroform for 30 min at room temperature. Finally, the coverslips were washed once with chloroform and once with 100% ethanol, then dried in a 70 °C oven for 1 h and stored in a drying basin.

### WGA conjugation

WGA (VECTOR, B-1025) was labeled with a copper-free click crosslinking agent using NHS-ester chemistry similar to previous reports^70,71^. DBCO-PEG5-NHS Ester (Kerafast, FCC310) was diluted to a concentration of 10 mM in anhydrous dimethyl sulfoxide (DMSO). Overall, 2.7 μL of the solution was then combined with 100 μL of 2 mg/mL WGA in DPBS. After 1 h at room temperature the reaction was terminated via an Amicon Ultra-0.5 Centrifugal Filter Unit (Millipore, UFC501024) at 14,000× *g* for 15 min, and then 1,000× *g* for 2 min to collect the DBCO labeled WGA. On average, the procedure produced ~2 DBCOs per WGA. Oligonucleotide probes, ordered from IDT and diluted to 100 μM in DPBS, contained the desired sequence (named OS_61r, /5Acryd/CGGTACGCACTTCCGTCGACGCAATAGCTC/3AzideN/) as well as a 5′-acrydite, to allow cross-linking to the PA gel, and a 3′-azide, to allow cross-linking to the DBCO-labeled WGA. A total of, 20 μL of the oligonucleotide was added to 100 μL of the DBCO-labeled WGA at a final concentration of ~2 mg/mL. This reaction was incubated at 4 °C for at least 12 h. The residual oligonucleotides were washed away from samples during primary probe staining.

### Primary probe staining

The primary probe staining procedure was similar to those in previous reports^27–29^. Tissue sections were stained with oligo-conjugated WGA in 1× hank’s balanced salt solution (HBSS) buffer for 20 min at 37 °C. Samples were post-fixed in 4% PFA for 10 min. Tissue sections were permeabilized using 0.5% Triton in DPBS for 20 min at room temperature before primary probe staining. Tissue samples were incubated for 5 min in pre-hybridization buffer composed of 50% (vol/vol) formamide and 2 mM Ribonucleoside vanadyl complexes (Sigma-Aldrich, R3380) in 2× saline sodium citrate (2× SSC) (Invitrogen, AM9765). Tissue samples were then stained with primary probes in primary hybridization buffer, containing 24-28 μM primary probes, 50% (vol/vol) formamide, 0.1% yeast tRNA (Invitrogen, 1885325), 1% (vol/vol) murine RNase inhibitor (NEB, M0314S), 10% (wt/vol) dextran sulfate (Millipore, S4030), and 2 μM anchor probe (a 15-nt sequence of alternating dT and thymidine-locked nucleic acid with a 5′ -acrydite modification) in 2× SSC, in a humidity chamber at 37 °C for 24 h. After staining, samples were washed for 15 min with 2× SSCT (2× SSC, 0.1% (vol/vol) Tween 20) twice at 60 °C and then once for 15 min at room temperature.

### Tissue embedding and clearing

To anchor RNAs and the oligos conjugated with WGA, the primary probe stained samples were embedded in 8% PA gels^29^. Briefly, a 1:30,000 dilution of 0.1-μm-diameter carboxylate-modified yellow-green fluorescent beads (Invitrogen, F8800) in 2× SSC containing 1% (vol/vol) VRC (NEB, S1402) was first incubated with the stained samples for 10 min at room temperature. Then, the samples were washed for 2 min with a de-gassed PA gel solution, containing 8% (vol/vol) 19:1 acrylamide/bis-acrylamide (Bio-Rad, 1610144), 60 mM Tris·HCl pH 8, 0.3 M NaCl, 0.03% (wt/vol) ammonium persulfate (Sigma, A3678-25G), and 0.15% (vol/vol) TEMED (AMERICANBIO.COM, AB02020-00050). To cast a thin PA gel film on the tissue samples, 50 μL of this PA gel solution was added to the surface of a glass plate that had been pretreated for 10 min with 1 mL GelSlick solution (Lonza, 50640). The sample coverslip was washed with PA gel solution and the excess solution on the coverslip was gently removed by dipping the edge of the coverslip on a Kimwipe tissue. Then, the sample coverslip was inverted and placed onto this 50 μL droplet to form a thin layer of PA gel between the coverslip and the glass plate. The PA gel was cast for 1.5 h at room temperature. The coverslip was then gently separated from the glass plate and incubated in digestion buffer for 12 h in a humid 37 °C incubator. The digestion buffer contained 2× SSC, 2% (vol/vol) SDS, and 1% (vol/vol) proteinase K (ThermoFisher, AM2548). The tissue was then washed with 2× SSC supplemented with 1% Murine RNase inhibitor on a shaker, three times each for 15 min.

### MERFISH imaging

After primary probe hybridization and tissue clearing, the samples were sequentially hybridized with different secondary probes, imaged and washed as previously reported^27–29^. We used Alexa Fluro 750/647-conjugated 20-nt secondary probes with sequences complementary to the readout regions of the primary probes (The secondary probe sequences are the same as previously reported)^28,29^ and an ATTO 565-conjugated 30-nt probe (OS_61, /5ATTO565N/AGAGCTATTGCGTCGACGGAAGTGCGTACCG) with the complementary sequence of the WGA-conjugated oligonucleotide (OS_61r). To label the cell membrane, we hybridized OS_61 (6 nM probes) in secondary hybridization buffer (2× SSC, 20% (vol/vol) ethylene carbonate (Sigma-Aldrich, E26258), 0.05% murine RNase inhibitor) to the samples at room temperature on a shaker for 15 min. Then we used a Bioptech’s FCS2 flow chamber and a home-built fluidics system to perform automatic buffer exchange during the multiple rounds of secondary hybridization. For each round of secondary hybridization, the samples were first incubated with the secondary hybridization buffer with 3 nM appropriate secondary probes for 10 min. We then sequentially washed the samples with 2 mL secondary wash buffer (20% (vol/vol) ethylene carbonate in 2× SSC) for 5 min and 2 mL imaging buffer with an oxygen scavenging system (50 mM Tris-HCl pH 8.0, 10% (wt/vol) glucose (Sigma, G8270), 2 mM Trolox (Sigma-Aldrich, 238813), 0.5 mg/mL glucose oxidase (Sigma-Aldrich, G2133), 40 μg/mL catalase (Sigma-Aldrich, C30), 0.05% (vol/vol) murine RNase inhibitor in 2× SSC) for 5 min. The imaging buffer in the input tube of the fluidics system was covered with a layer of mineral oil (Sigma, 330779) to prevent continuous oxidation. Next, the samples were imaged at multiple fields of view. For each field of view, we took z-stack images with 750, 647, 560, and 488 nm laser illuminations. The range of the z-stacks was 9 μm overall, with 16 steps of 0.6 μm intervals. After each round of imaging, we flowed secondary probe removal buffer (65% formamide in 2× SSC) for 10 min to wash out secondary probes.

### Imaging system

For imaging, we used a home-built microscope with a Nikon Ti2-U body, a Nikon CFI Plan Apo Lambda 60× Oil (NA1.40) objective lens, and an active auto-focusing system as described before^72^. A 750-nm laser (2RU-VFL-P-500-750-B1R, MPB Communications) was used to excite and image Alexa Fluor 750 on secondary probes. A 647-nm laser (2RU-VFL-P-1000-647-B1R, MPB Communications) was used to excite and image Alexa Fluor 647 on secondary probes. A 560-nm laser (2RU-VFL-P-1000-560-B1R, MPB Communications) was used to excite and image ATTO 565 on cell membrane probe OS_61. A 488-nm laser (2RU-VFL-P-500-488-B1R, MPB Communications) was used to excite and image the yellow-green fiducial beads for drift correction. The four laser lines were directed to the samples using a multi-band dichroic mirror (488/561/647/752rpc-UF2, Chroma) on the excitation path. On the emission path, we had a multi-band emission filter (488/561/647–656/752 m, Chroma) and a Hamamatsu Orca Flash 4.0 V3 camera. The pixel size of our system was measured to be 107.9 nm per pixel. To automatically scan and image multiple fields of view, we used a computer-controlled motorized x-y sample stage (SCAN IM 112 × 74, Marzhauser).

### scRNA-seq

For sorted HSC scRNA-seq, fetal liver tissues were isolated from E14.5 C57BL/6 pregnant mice and in cold 1× PBS dissociated into single cell suspension by pipetting up and down through 15 mL pipette. Red blood cells were removed using 1× RBC lysis buffer (diluted into 1× in deionized water from 10× RBC lysis buffer, Santa Cruz Biotechnology, sc-296258) at room temperature for 5 min. Cells were incubated for 5 min at room temperature and mixed constantly by inverting the tube. After that, cells were centrifuged for 5 min and then the supernatant was carefully removed without disturbing the pellet cells. Cells were resuspended in cold 1× PBS with 0.5% BSA, passed through a 40 μm strainer (Falcon, 352235). Fetal liver cells were used for FACS to isolate HSCs as described below. For whole fetal liver scRNA-seq, fetal liver tissues were isolated from E14.5 C57BL/6 pregnant mice and digested by dissociation enzyme (Accutase, A1110501) for 10 min at room temperature. Red blood cells were removed using 1× RBC lysis buffer (Santa Cruz Biotechnology, sc-296258) at 4 °C. Then, cells were passed through a 40 μm strainer (Falcon, 352235) and re-suspended in cold DPBS with 0.04% (wt/vol) BSA. We measured cell viability with a TC20TM Automated Cell Counter (Bio-Rad), and then took 8,000 cells for 10× library preparation using Chromium Single Cell 3′ Reagent Kits (v2). The libraries were run on an Illumina HiSeqX10 as 150 bp paired-end reads and occupied one full lane.

### FACS sorting of fetal liver HSC

HSCs were sorted under the same condition as previously described^73^. Before sorting HSCs, we added Biotin Lineage depletion cocktail (anti-Mac1, anti-CD3ε, anti-B220, anti-Gr-1, anti-CD11b, and anti-Ter119) and streptavidin Particles Plus-DM (BD IMag™, mouse hematopoietic progenitor (stem) cell enrichment set). Lineage positive cells were depleted by transferring cells to a magnetic stand. Enriched lineage negative cells were further stained with antibodies (LSK CD150^+^CD34^−^CD48^−^Flt3^−^) listed in Supplementary Table S5. HSCs were sorted with FACS AriaII (BD Biosciences) from the Yale Cell Sorter Core Facility.

### Processing of scRNA-seq data

The FASTQ format results were analyzed using Cell Ranger (https://support.10xgenomics.com/single-cell-gene-expression/software/pipelines/latest/using/tutorial_ov) for sample demultiplexing, barcode processing, and gene counting. The reference genome mm10/GRCm38 build by 10× genomics was used to align inserted cDNA. According to cell barcode and unique molecular identifiers, 9,448 single fetal liver cells and 8,613 single fetal liver HSCs were identified. Further analysis was performed using the Seurat R package^74^, including quality filtering, the identification of highly variable genes, dimensionality reduction, standard unsupervised clustering, and the discovery of DEGs. We removed low-quality cells that had fewer than 500 detected genes and genes expressed in less than three cells, as well as cells with more than 10% of the transcripts coming from mitochondrial genes. Next, we normalized the data by the total expression, multiplied the data by the scale factor 10,000, added 1 and log-transformed the result. To remove batch effects, we used the Seurat alignment method CCA (canonical correlation analysis) for data integration. We used 2,500 anchor features for the canonical correlation analysis. The subspaces were aligned on the basis of the first 30 canonical correlation vectors to generate a combined aligned dataset to use for further integrative analysis. To find out HSC markers at the transcriptome level, we further reduced dimensionality of the entire integrated dataset using Uniform Manifold Approximation and Projection (UMAP), whereby the major cell type identification was based on the Mouse Cell Atlas^30^. We used the cell type terms and the corresponding marker genes reported in the associated web database: http://bis.zju.edu.cn/MCA/gallery.html?tissue=Fetal-Liver. To analyze the GO of hematopoietic cells, we calculated the DEGs using Seurat, which was used as an input for GO analysis with Clusterprofiler^75^. For the RNA velocity analysis, we first generated the spliced and unspliced RNA loom file using velocyto, and then performed the velocity analysis using velocity.R in Seurat, and the cells were visualized using UMAP. For the cellphone analysis, we first converted the mouse genes to human homologous genes using biomaRt^76^, and then analyzed the interaction between different cell types using the integrated dataset via CellphoneDB^33^.

### Bulk RNA sequencing

Total RNA was extracted from E14.5 fetal liver using RNeasy Mini Kit (QIAGEN, 74104). mRNA was isolated using the Dynabeads mRNA Purification Kit (THERMOFISHER, 61006). The cDNA libraries were produced with a NEBNext^®^ Ultra™ II RNA Library Prep Kit for Illumina^®^ (NEB, E7770S) and NEBNext^®^ Multiplex Oligos for Illumina^®^ (NEB, E7335S) according to the manufacturer’s description. Finally, the cDNA libraries were sequenced on an Illumina HiSeqX10 platform with 150 bp paired-end reads.

### MERFISH data processing

All MERFISH analyses in this study were performed with MATLAB (MATLAB, 9.6.0.1150989 (R2019a) Update 4). To efficiently analyze MERFISH images, we implemented a pixel-based MERFISH analysis pipeline similar to that introduced in a previous report^27–29^. We corrected the sample drift between different rounds of MERFISH imaging based on the fiducial beads and decoded the RNA signals according to each drift-corrected RNA image. We matched the decoded RNA signals with our MERFISH codebook (Supplementary Table S1). The molecular counts detected by MERFISH for each RNA species were compared with the FPKMs from bulk RNA-seq by Pearson correlation analysis. MERFISH data were used for further analysis only when the correlation coefficient was greater than 0.7. When correlating MERFISH copy numbers and bulk RNA-seq FPKMs, we found the expression of a small set RNA species (eight RNA species, including *Angpt2*, *Ddx4*, *Dkk1*, *Gata3*, *Hoxb5*, *Selp*, *Wnt2*, *Wnt4*) were not consistent with bulk RNA-seq data. These outlier genes were removed before the next steps of analyses. The displayed correlation analysis of FPKM vs total RNA copy numbers for WT (Fig. 1e) and the correlation of total RNA copy numbers from two MERFISH experiments (Fig. 1f) were from one of the biological replicates.

We segmented the tissue section into single cells based on the WGA labeling pattern using a watershed algorithm, and assigned RNA counts into individual cells based on cell boundaries to generate the gene by cell expression matrix as previously described^77^. When analyzing single-cell RNA data, we removed “cells” larger than 20,000 pixels or less than 2,500 pixels in area as these were usually empty regions or non-cell particles in the tissue section. We removed partial cells overlapping the edges of each field of view. We removed cells with < 10 detected RNA molecules to ensure high quality in the cell type identification analyses. To cluster cells based on the RNA copy number profiles, we applied the Louvain–Jaccard clustering algorithm^78^, and visualized the cell clusters with UMAP. We identified the cell types of the clusters based on the RNA expression patterns in the clusters. The displayed MERFISH single cell cluster result (Fig. 1c) was from one of the WT biological replicates.

To analyze the HSC niches in situ, we determined HSCs according to the expression of *Mecom* and *Kit*. First, we calculated the 99.95 percentile of cells based on the RNA copy numbers of *Mecom* and set this copy number as the *Mecom* threshold, where cells with a greater *Mecom* RNA copy number than this threshold were considered to be potential HSCs. Then, we identified HSCs from potential HSCs whose copy numbers of *Kit* were equal to or greater than 1. After the HSC identification, we defined a niche by selecting cells surrounding an HSC within a radius of 20 μm. For all the DEGs and gene co-expression analyses, we first normalized the expression of each gene in each cell: the copy number of each RNA species in the cell was divided by the copy number of all labeled RNA species in the cell, and was then multiplied by 1,000. The normalized copy numbers are proportional to the fractions of total RNA counts that belong to each RNA species within the cells.

To calculate the gene expression enrichment for each cell type, we first calculated the average gene expression profile of each cell type, and then divided the profile by the average gene expression profile of the entire cell population. The result was then log_2_ transformed. For better display, we further clustered and reordered the niche genes, Wnt genes, Notch genes, and other genes using hierarchical clustering. The displayed log_2_ gene expression enrichment matrices for WT and *Tet2*^−*/*−^ (Fig. 3a, b) were derived from all biological replicates.

In the gene-gene correlation analysis, we calculated the correlation coefficients between the expression levels of pairs of genes among individual cells in the HSC niche. The displayed correlation coefficients for WT and *Tet2*^−*/*−^ niche (Fig. 5c, d) were derived from the niche cells from all biological replicates. When a niche gene was correlated with multiple marker genes of the same cell type, we displayed the highest correlation coefficient. The *P* values for testing the multiple hypotheses were adjusted using false discovery rate. All significant correlations, defined as correlations with correlation coefficients *R* > 0.1 and adjusted *P* < 0.05, were listed in the Supplementary Table S3.

The enrichment of pairs of cell types in spatial proximity was calculated by first measuring the probability of finding each pair of cell types within 20 μm distance, and then normalizing this probability to the control probability derived from randomly pairing cell types given their abundance in the whole population. The *P* values of the enrichment were calculated by chi-square test and corrected using false discovery rate. The enrichment folds were log_2_ transformed for display. The displayed enrichment of pairs of cell types in spatial proximity for WT and *Tet2*^−*/*−^ (Fig. 2a–f) were from all the biological replicates in each case. To determine reproducible enrichment observations (cyan dots in Fig. 2a–d), an enrichment needs to be reproducibly significant (*P* < 0.01) in three individual biological replicates (out of four total biological replicates for WT and three total biological replicates for *Tet2*^−*/*−^).

All “Adjusted *P* value” shown in this paper were corrected for multiple hypothesis testing using false discovery rate. To analyze single cell data for reproducibility, we divided the data into two replicate groups (each group contains multiple independent biological replicates) for WT and *Tet2*^*−*^^*/*−^, and performed the DEGs analysis for each replicate group. Only genes that were significant in the total data (with a fold change ≥ 2 one way or the other, and *P* < 0.05) and in both replicate groups (with a fold change trend consistent with the total data and *P* < 0.1) were reported as significant DEGs. Any genes that were not significant in either of the replicate groups were disregarded as unreproducible findings. The displayed DEG volcano plots show results from all biological replicates.

## Supplementary information

Table S1

Table S2

Table S3

Table S4

Table S5

Fig S2

Fig S3

Fig S4

Fig S5

Fig S6

Fig S7

Fig S8

Fig S9

Fig S10

Fig S1

## Data Availability

Raw imaging data are available from S.W. on request. Analyzed imaging data are available at: https://campuspress.yale.edu/wanglab/HSCMERFISH. Bulk RNA sequencing data from this work are available at: GSE172128. Single cell RNA sequencing data from this paper are available at: GSE172127.

## Code availability

Data were collected using open source python codes from https://github.com/ZhuangLab/storm-control. All MATLAB programs and R scripts for data analysis are available at https://campuspress.yale.edu/wanglab/HSCMERFISH.
