# Supplementary material for: Spatial transcriptome profiling by MERFISH reveals fetal liver hematopoietic stem cell niche architecture
Source: Cell Discov. 2021 Jun 29;7:47. doi: 10.1038/s41421-021-00266-1 (PMC8238952; doi:10.1038/s41421-021-00266-1)
Supplement: Supplementary file 6 — Fig S2 [file 41421_2021_266_MOESM6_ESM.pdf]

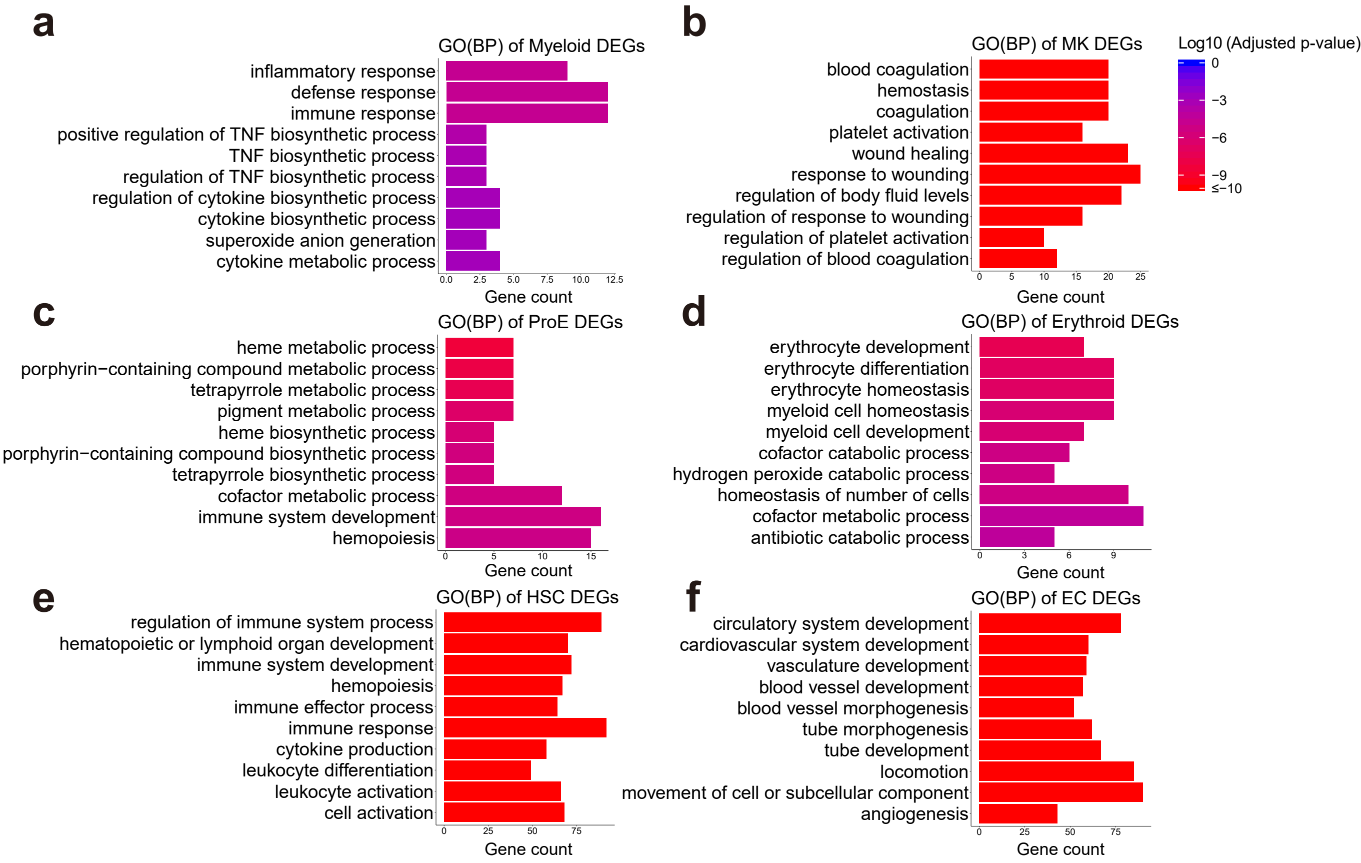

Supplementary Fig. S2 Gene ontology (biological processes) of fetal liver cells. a-f The gene ontology (GO) analysis of myeloid cell (**a**), megakaryocyte (MK) (**b**), erythroid progenitor (ProE) (**c**), erythroid cell (**d**), hematopoietic stem cell (HSC) (**e**) and endothelial cell (EC) (**f**), respectively. The differentially expressed genes (DEGs) were calculated through single cell RNA sequencing analysis by Seurat (adjusted p-value < 0.05; Wilcoxon rank-sum test with Bonferroni correction; fold change > 2). For each GO analysis, we show the top 10 categories of biological processes (BP) of the DEG sets. p-values were log10 transformed, the color scale for (**a-f**) is shown on the top right. TNF, tumor necrosis factor.
