# Supplementary material for: Spatial transcriptome profiling by MERFISH reveals fetal liver hematopoietic stem cell niche architecture
Source: Cell Discov. 2021 Jun 29;7:47. doi: 10.1038/s41421-021-00266-1 (PMC8238952; doi:10.1038/s41421-021-00266-1)
Supplement: Supplementary file 7 — Fig S3 [file 41421_2021_266_MOESM7_ESM.pdf]

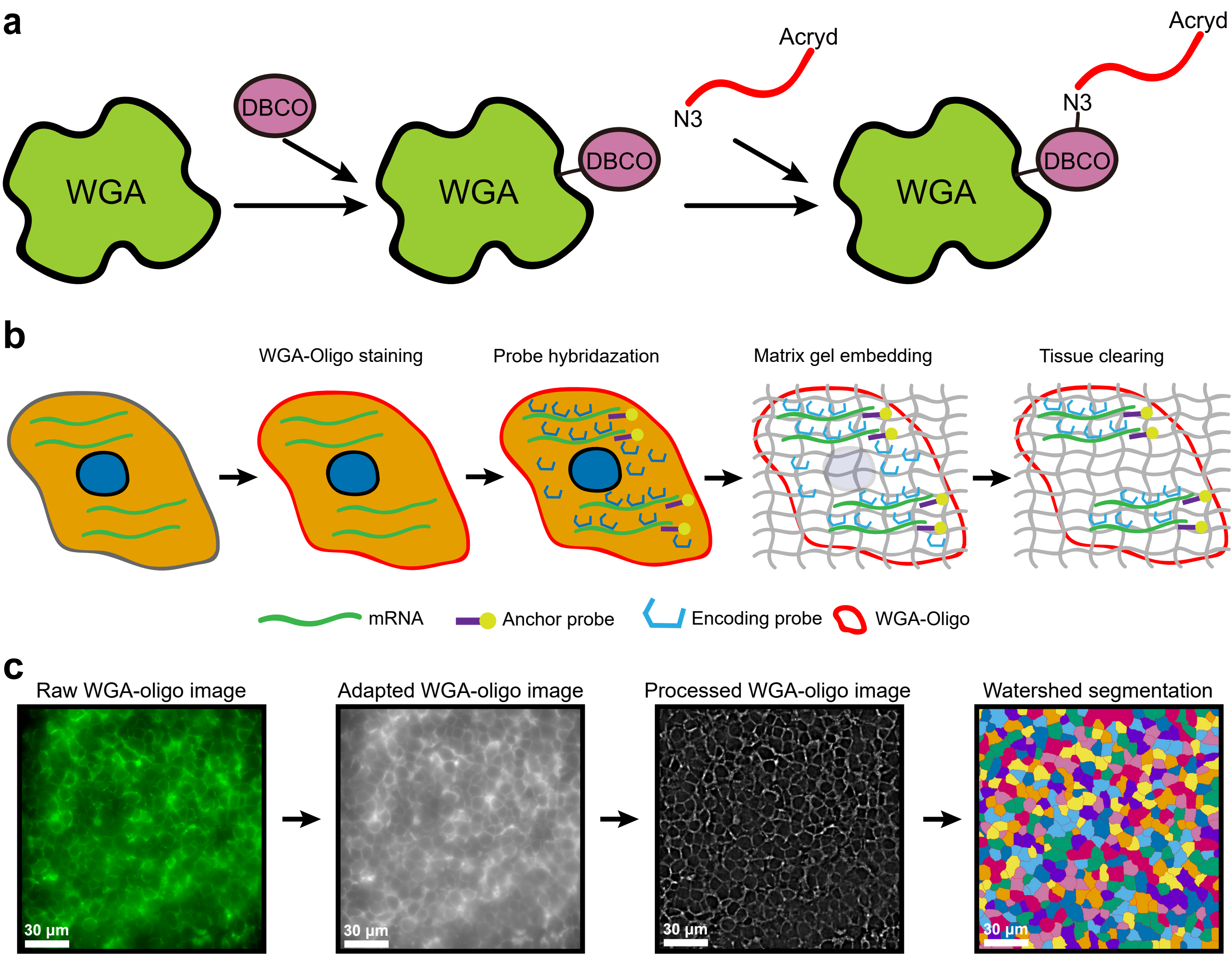

Supplementary Fig. S3 Schematics of cell membrane labeling strategy. a Schematics of the WGA-oligo conjugation. WGA is conjugated with DBCO, which reacts with the 3’-azide groups on oligos. The 5’-acrydite groups on oligos enable the crosslinking of the WGA-oligo conjugates into a polyacrylamide gel. **b** Schematics of the membrane staining, probe hybridization, matrix gel embedding and tissue clearing for MERFISH imaging. **c** Raw image and processed images of oligo-conjugated WGA.
