# Supplementary material for: Spatial transcriptome profiling by MERFISH reveals fetal liver hematopoietic stem cell niche architecture
Source: Cell Discov. 2021 Jun 29;7:47. doi: 10.1038/s41421-021-00266-1 (PMC8238952; doi:10.1038/s41421-021-00266-1)
Supplement: Supplementary file 8 — Fig S4 [file 41421_2021_266_MOESM8_ESM.pdf]

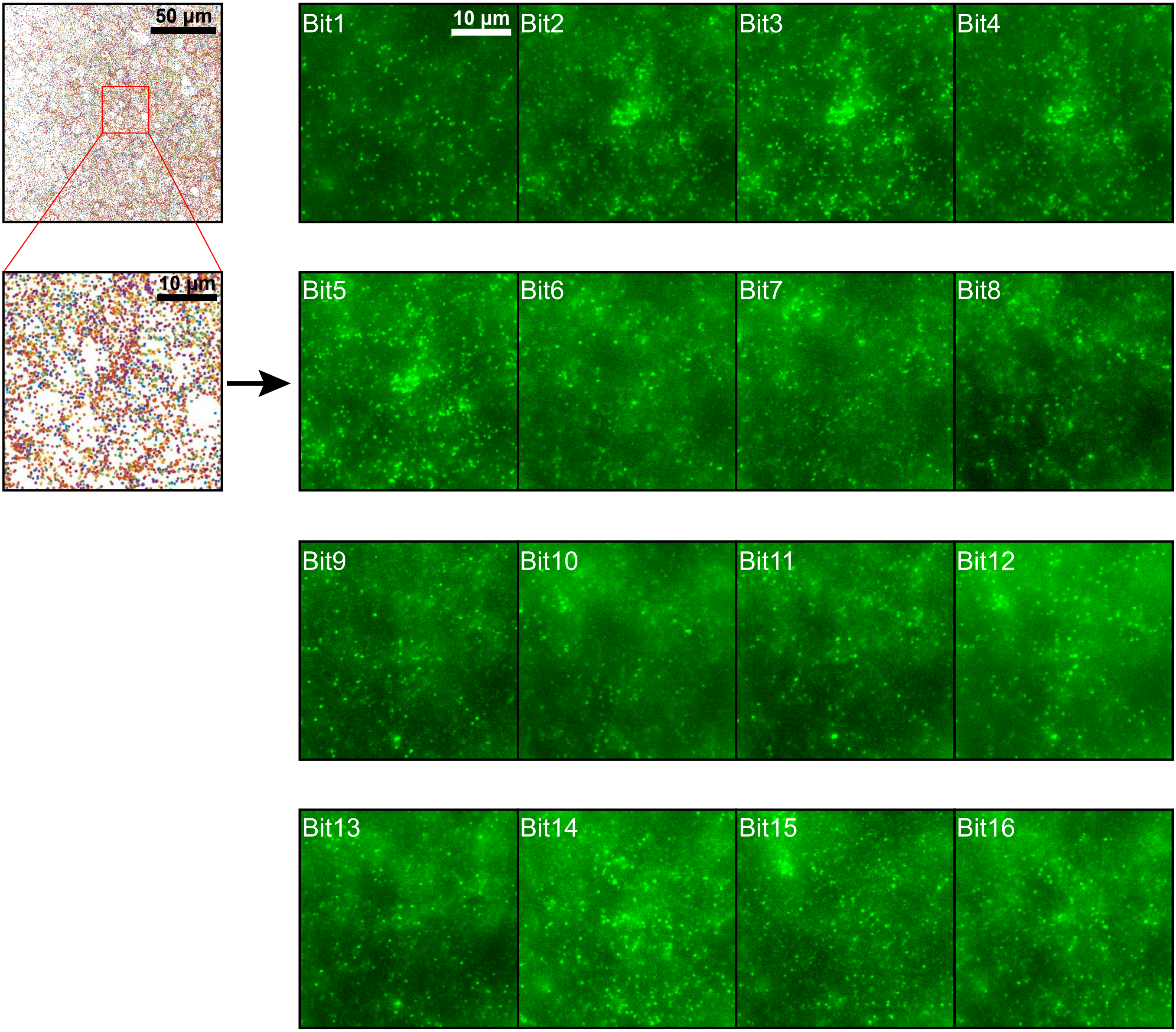


**Supplementary Fig. S4 Analyzed and raw images of RNA molecules in MERFISH imaging.** Images shown here are from a single z position in a 3D image stack. The left two panels show analyzed RNA molecules that are pseudo-colored by their gene identities. The right 16 panels show the raw RNA images in the same imaging field.
