# Supplementary material for: Spatial transcriptome profiling by MERFISH reveals fetal liver hematopoietic stem cell niche architecture
Source: Cell Discov. 2021 Jun 29;7:47. doi: 10.1038/s41421-021-00266-1 (PMC8238952; doi:10.1038/s41421-021-00266-1)
Supplement: Supplementary file 9 — Fig S5 [file 41421_2021_266_MOESM9_ESM.pdf]

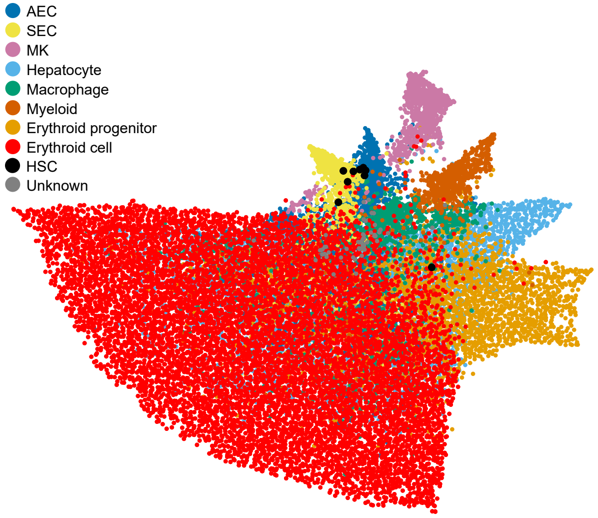

Supplementary Fig. S5 UMAP plot of MERFISH dataset of WT fetal liver. N = 40,864 cells.

The identified HSCs are indicated with black dots (N = 8 cells).
