# Supplementary material for: Spatial transcriptome profiling by MERFISH reveals fetal liver hematopoietic stem cell niche architecture
Source: Cell Discov. 2021 Jun 29;7:47. doi: 10.1038/s41421-021-00266-1 (PMC8238952; doi:10.1038/s41421-021-00266-1)
Supplement: Supplementary file 10 — Fig S6 [file 41421_2021_266_MOESM10_ESM.pdf]

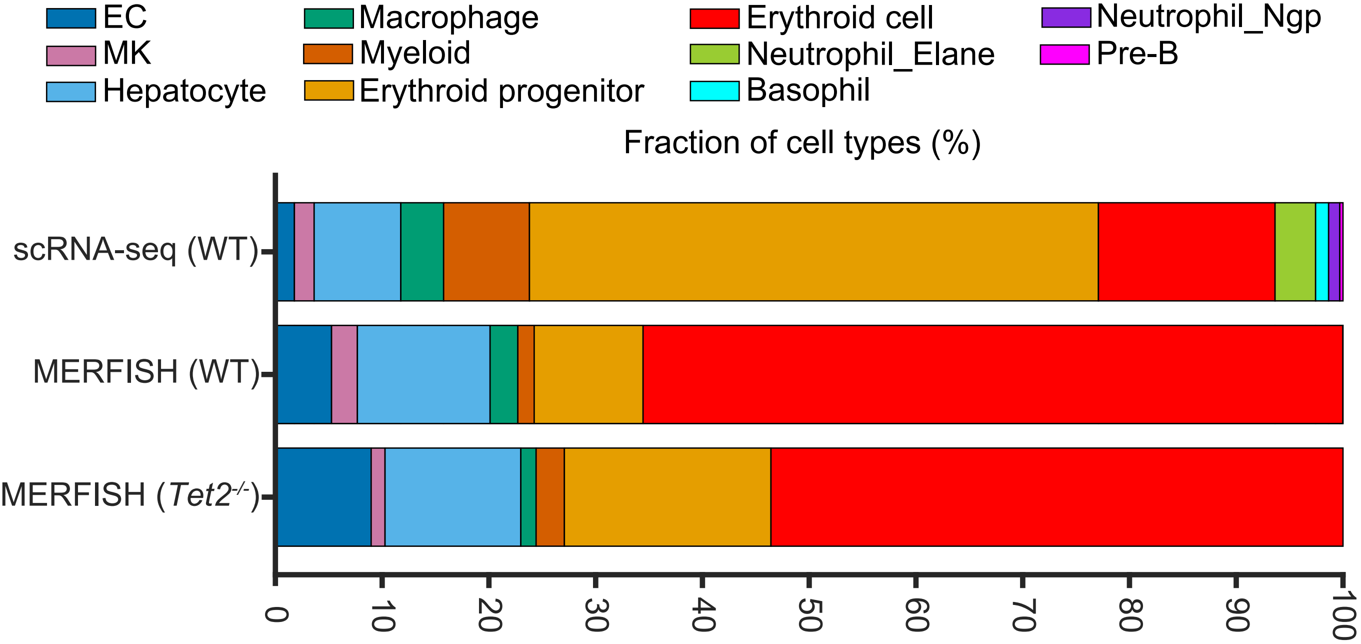


**Supplementary Fig. S6 The proportion of cell types identified in scRNA-seq and MERFISH in WT and *Tet2*$^{-/-}$ fetal livers.**
