# Supplementary material for: Spatial transcriptome profiling by MERFISH reveals fetal liver hematopoietic stem cell niche architecture
Source: Cell Discov. 2021 Jun 29;7:47. doi: 10.1038/s41421-021-00266-1 (PMC8238952; doi:10.1038/s41421-021-00266-1)
Supplement: Supplementary file 11 — Fig S7 [file 41421_2021_266_MOESM11_ESM.pdf]

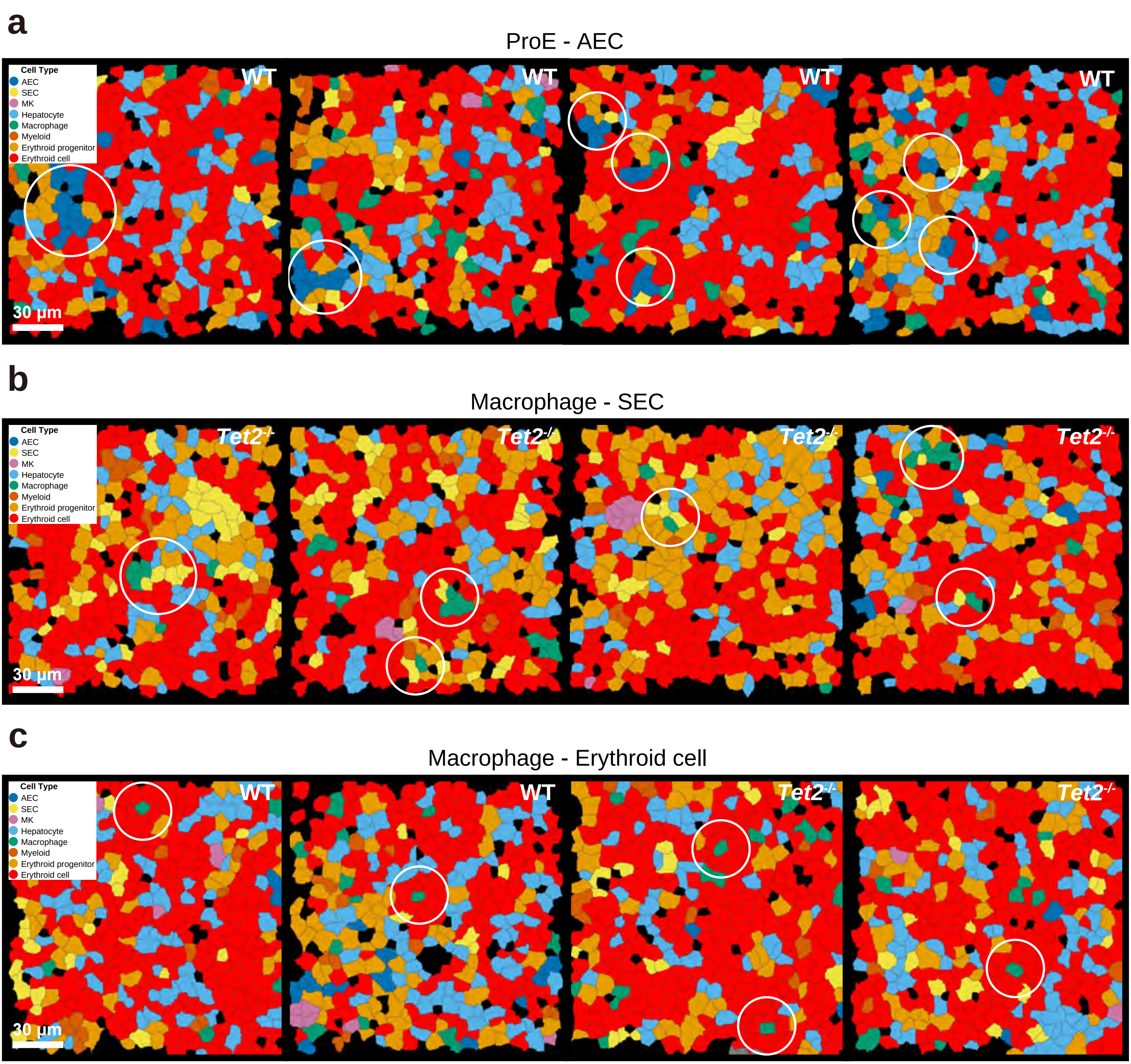

Supplementary Fig. S7 Examples of cell type pairing in spatial neighborhood. a Cell type plots of ProE (light brown) paired with AECs (dark blue) in WT fetal liver. **b** Cell type plots of Macrophages (green) paired with SECs (yellow) in *Tet2*$^{-/-}$ fetal liver**. c** Cell type plots of a central macrophage (green) in an erythroid island (red) in WT and *Tet2*$^{-/-}$ fetal liver. ProE, erythroid progenitor; AEC, arterial endothelial cell; SEC, sinusoidal endothelial cell.
