# Supplementary material for: Spatial transcriptome profiling by MERFISH reveals fetal liver hematopoietic stem cell niche architecture
Source: Cell Discov. 2021 Jun 29;7:47. doi: 10.1038/s41421-021-00266-1 (PMC8238952; doi:10.1038/s41421-021-00266-1)
Supplement: Supplementary file 12 — Fig S8 [file 41421_2021_266_MOESM12_ESM.pdf]

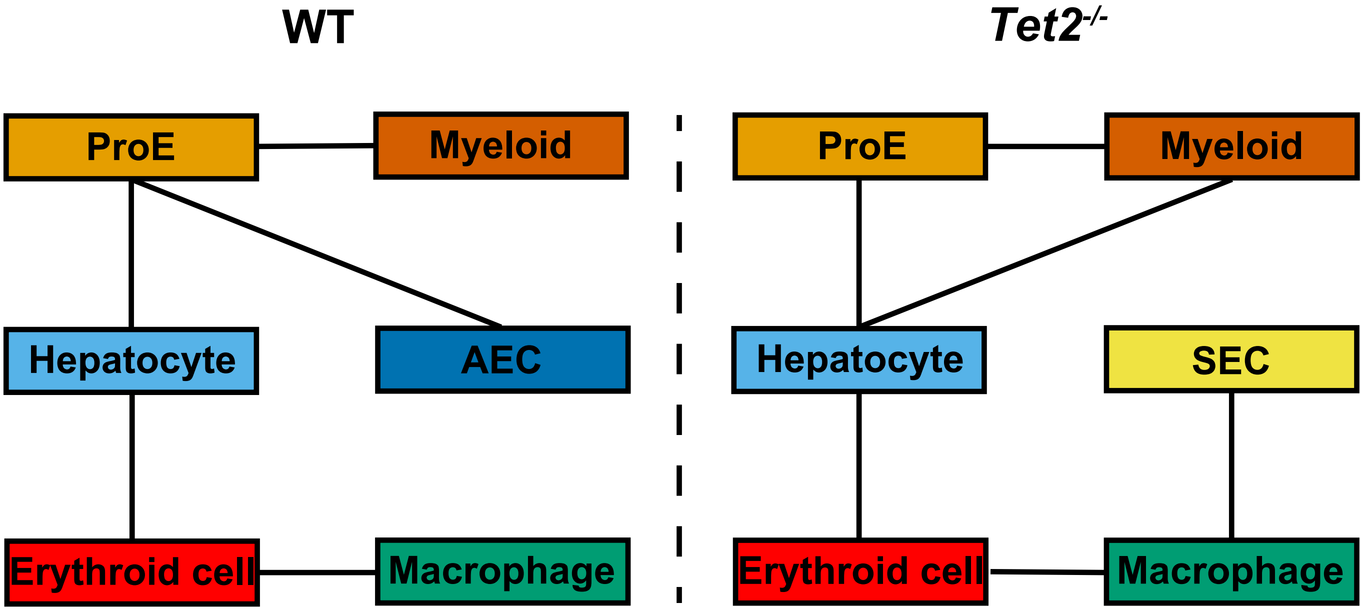

Supplementary Fig. S8 The diagram of significantly enriched cell-cell interactions in WT and *Tet2*$^{-/-}$ fetal livers. AEC, arterial endothelial cell; SEC, sinusoidal endothelial cell; ProE, erythroid progenitor.
