# Supplementary material for: Spatial transcriptome profiling by MERFISH reveals fetal liver hematopoietic stem cell niche architecture
Source: Cell Discov. 2021 Jun 29;7:47. doi: 10.1038/s41421-021-00266-1 (PMC8238952; doi:10.1038/s41421-021-00266-1)
Supplement: Supplementary file 13 — Fig S9 [file 41421_2021_266_MOESM13_ESM.pdf]

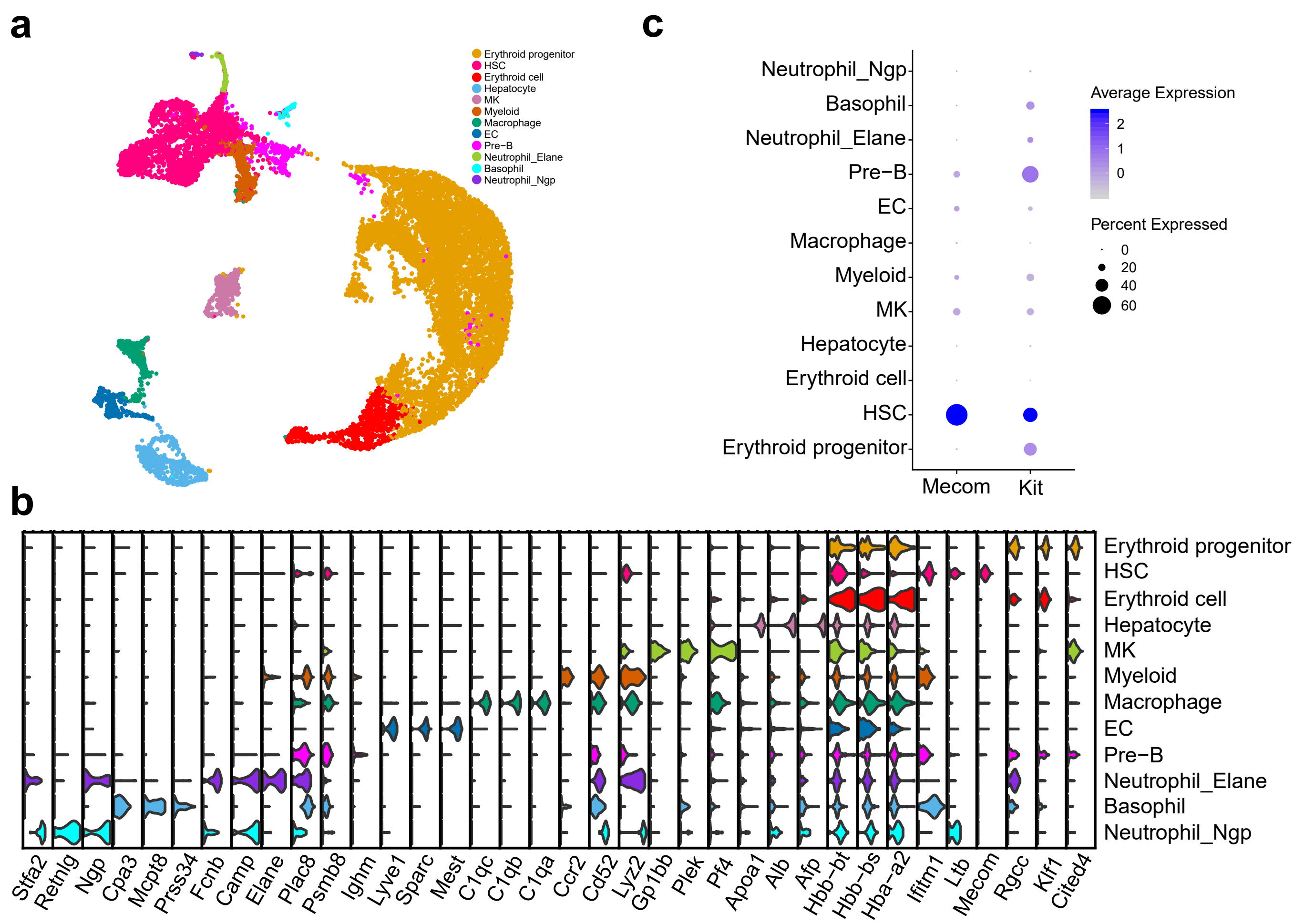

Supplementary Fig. S9 The expression patterns of *Mecom* and *Kit* in merged single cell RNA-seq data. a Merged UMAP plot of scRNA-seq performed on whole fetal liver cell population (N = 7,635 cells) and sorted HSCs (N = 6,097 cells). Color code for cell types is shown on the right. **b** Violin plot of cell type marker gene expression in different cell types from merged single-cell RNA-seq data. X axis shows normalized expression data ranging from 0 to 6 for each gene (See Methods for the normalization procedure). We attained 12 clustered cell types from the merged single-cell data. Specific marker genes shown are: *Cited4*, *Klf1* and *Rgcc* for erythroid progenitor, *Mecom*, *Ltb* and *Ifitm1* for HSC, *Hba-a2*, *Hbb-bs* and *Hbb-bt* for erythroid cell, *Afp*, *Alb* and *Apoa1* for hepatocyte, *Pf4*, *Plek* and *Gp1bb* for megakaryocyte (MK), *Lyz2*, *Cd52* and *Ccr2* for myeloid, *C1qa*, *C1qb* and *C1qc* for macrophage, *Mest*, *Sparc* and *Lyve1* for endothelial cell (EC), *Ighm*, *Psmb8* and *Plac8* for pre-B cell, *Elane*, *Camp* and *Fcnb* for neutrophil_Elane, *Prss34*, *Mcpt8* and *Cpa3* for basophil, and *Ngp*, *Retnlg* and *Stfa2* for neutrophil_Ngp. **c** Dot plot of *Mecom* and *Kit* expression in merged single cell data. *Mecom* is highly expressed in HSC clusters and barely in EC cluster, while *Kit* is expressed in many clusters such as HSCs, erythroid progenitors and Pre-B cells, but shows near-zero expression in ECs. The colors of the dots indicate the average expression levels of the marker genes and the sizes of the dots indicate the percentages of cells in the cell type clusters that expressed the marker genes (color and size scales on the right).
