# Supplementary material for: Spatial transcriptome profiling by MERFISH reveals fetal liver hematopoietic stem cell niche architecture
Source: Cell Discov. 2021 Jun 29;7:47. doi: 10.1038/s41421-021-00266-1 (PMC8238952; doi:10.1038/s41421-021-00266-1)
Supplement: Supplementary file 14 — Fig S10 [file 41421_2021_266_MOESM14_ESM.pdf]

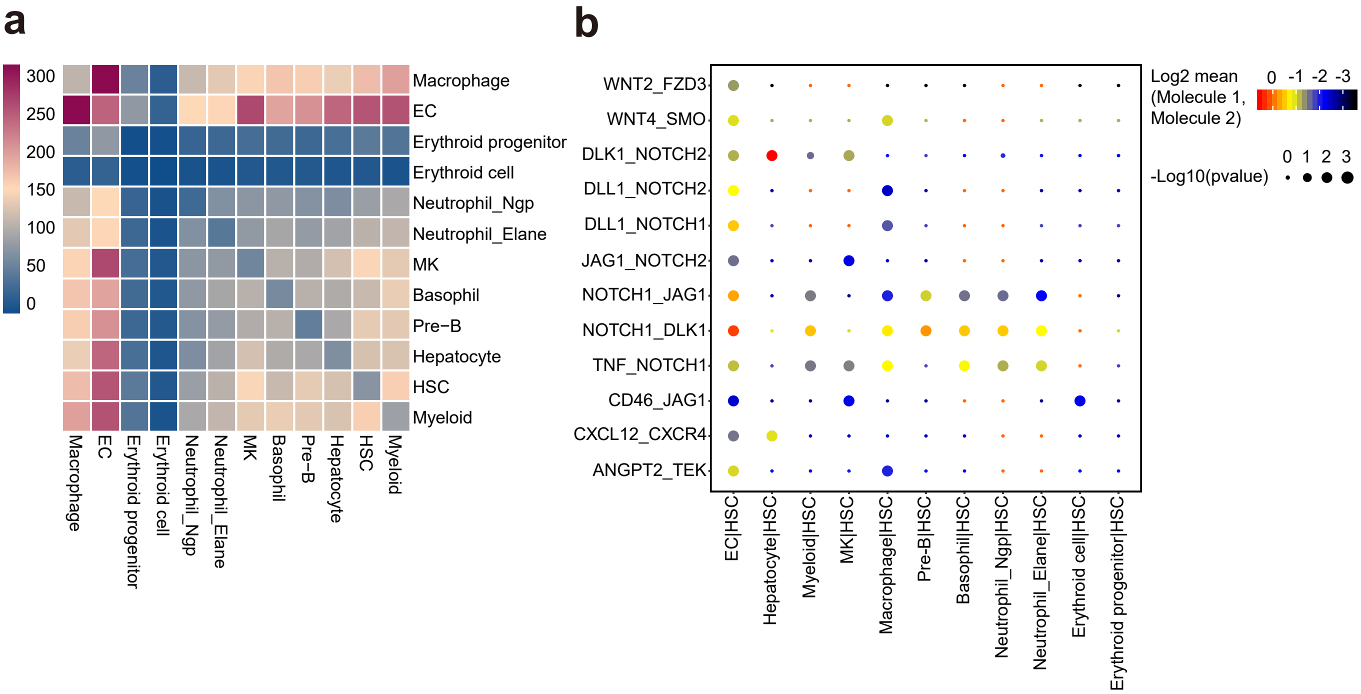

Supplementary Fig. S10 Cell-cell interaction analysis using the CellPhoneDB in single cell RNA-seq datasets. a Cell-cell communication analysis by the CellphoneDB showing the total number of interactions between cell types based on the known ligand and receptor pairs expressed in different cell types. **b** Selected ligand-receptor interactions analysis by the CellPhoneDB. p-values are indicated by circle size, with the scale on right. Specific ligand-receptor pairs are listed on the left and the corresponding cell types in which they are expressed are listed at the bottom. The means of the average expression level of interacting molecule 1 in cell type 1 and interacting molecule 2 in cell type 2 are indicated by different colors shown on the right.
