# Supplementary material for: Spatial transcriptome profiling by MERFISH reveals fetal liver hematopoietic stem cell niche architecture
Source: Cell Discov. 2021 Jun 29;7:47. doi: 10.1038/s41421-021-00266-1 (PMC8238952; doi:10.1038/s41421-021-00266-1)
Supplement: Supplementary file 15 — Fig S1 [file 41421_2021_266_MOESM15_ESM.pdf]

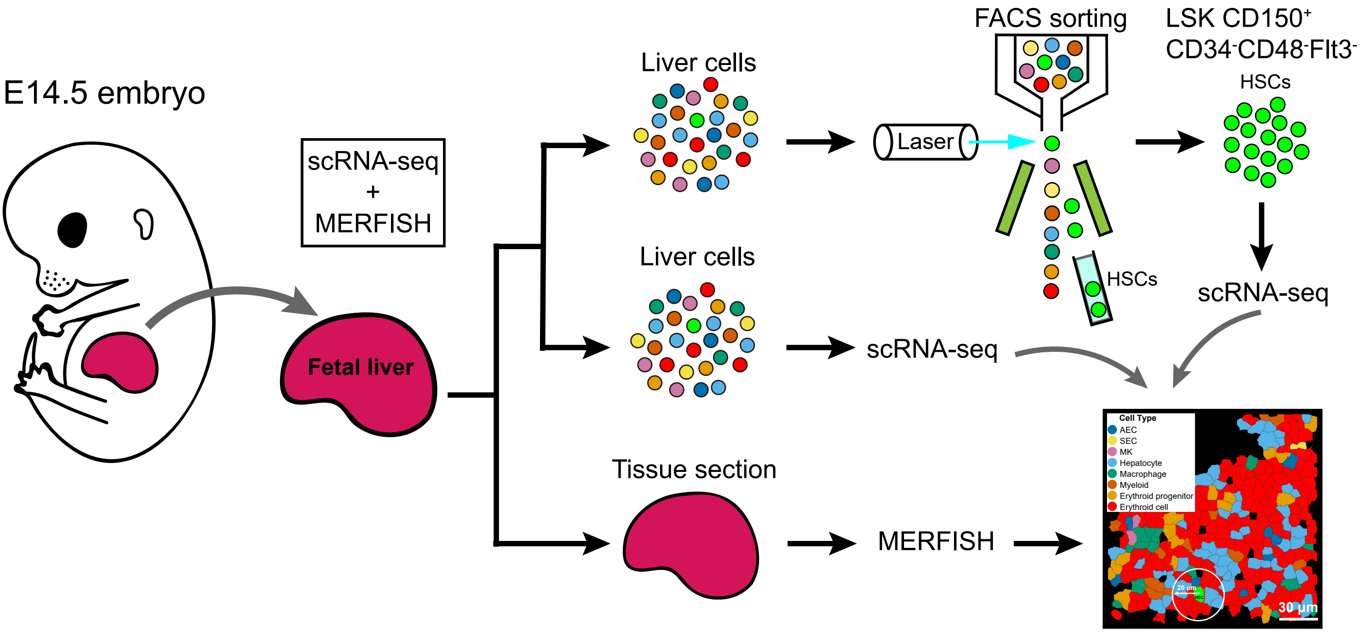

Supplementary Fig. S1 Schematic illustration of the experimental workflow. Fetal livers were dissected from E14.5 embryos. For single-cell RNA sequencing analysis (scRNA-seq), fetal liver cells are dissociated into single cells. Then cells are either sorted to enrich HSCs or not sorted to retain the whole population, and sequenced with the 10x Genomics scRNA-seq platform. For MERFISH analysis, we design probes targeting marker genes of different cell types and genes important for HSC and HSC niche functions, and analyze their spatial expression patterns in single cells in E14.5 fetal liver tissue sections.
